# ALKBH5‐mediated m^6^A demethylation ameliorates extracellular matrix deposition in cutaneous pathological fibrosis

**DOI:** 10.1002/ctm2.70016

**Published:** 2024-09-04

**Authors:** Ruoqing Xu, En Yang, Hsin Liang, Shenying Luo, Yunhan Liu, Yimin Khoong, Haizhou Li, Xin Huang, Yixuan Zhao, Tao Zan

**Affiliations:** ^1^ Department of Plastic and Reconstructive Surgery Shanghai Ninth People's Hospital Shanghai Jiao Tong University School of Medicine Shanghai P.R. China

**Keywords:** ALKBH5, ECM, hypertrophic scar, m^6^A modification

## Abstract

**Background:**

Elevated extracellular matrix (ECM) accumulation is a major contributing factor to the pathogenesis of fibrotic diseases. Recent studies have indicated that N6‐methyladenosine (m^6^A) RNA modification plays a pivotal role in modulating RNA stability and contribute to the initiation of various pathological conditions. Howbeit, the precise mechanism by which m^6^A influences ECM deposition remains unclear.

**Methods:**

In this study, we used hypertrophic scars (HTSs) as a paradigm to investigate ECM‐related diseases. We focused on the role of ALKBH5‐mediated m^6^A demethylation within the pathological progression of HTSs and examined its correlation with clinical stages. The effects of ALKBH5 ablation on ECM components were studied both in vivo and in vitro. Downstream targets of ALKBH5, along with their underlying mechanisms, were identified using integrated high‐throughput analysis, RNA‐binding protein immunoprecipitation and RNA pull‐down assays. Furthermore, the therapeutic potential of exogenous ALKBH5 overexpression was evaluated in fibrotic scar models.

**Results:**

ALKBH5 was decreased in fibroblasts derived from HTS lesions and was negatively correlated with their clinical stages. Importantly, ablation of ALKBH5 promoted the expression of COL3A1, COL1A1, and ELN, leading to pathological deposition and reconstruction of the ECM both in vivo and in vitro. From a therapeutic perspective, the exogenous overexpression of ALKBH5 significantly inhibited abnormal collagen deposition in fibrotic scar models. As determined by integrated high‐throughput analysis, key ECM components including COL3A1, COL1A1, and ELN are direct downstream targets of ALKBH5. By means of its mechanism, ALKBH5 inhibits the expression of COL3A1, COL1A1, and ELN by removing m^6^A from mRNAs, thereby decreasing their stability in a YTHDF1‐dependent manner.

**Conclusions:**

Our study identified ALKBH5 as an endogenous suppressor of pathological ECM deposition, contributing to the development of a reprogrammed m6A‐targeted therapy for HTSs.

## Graphical abstract

1. ALKBH5‐mediated m^6^A demethylation serves as an intrinsic brake in the regulation of the ECM in fibroblasts within hypertrophic scars.

2. Key ECM components, including COL3A1, COL1A1 and ELN are direct downstream targets of ALKBH5.

3. Exogenous overexpression of ALKBH5 significantly reversed the fibrotic phenotype both in vivo and in vitro.

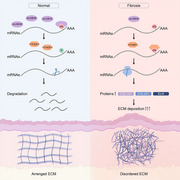

## 1 INTRODUCTION

The extracellular matrix (ECM) serves as the intercellular structural framework and is predominantly composed of fibrillar proteins (collagen fibres and elastic fibres).^1^ The ECM plays a pivotal role in maintaining tissue integrity, facilitating intercellular signalling, and governing the physiological status of the local microenvironment.^2^, ^3^ In particular, during the progression of injury repair and tissue remodelling, the dynamics of the ECM undergo qualitative and quantitative changes, ultimately influencing the activation and function of resident cells.^4^ The current prevailing view in the field indicates that the primary pathological process of hypertrophic scars (HTSs) involves the abnormal activity of fibroblasts producing excessive disorganised ECM components, leading to tissue stiffening in dermal fibrosis.^5^, ^6^, ^7^

Mechanistically, dynamic alterations occur in dermal ECM components during scar formation^6^: the cytoskeletal protein αSMA serves as a marker for the activation of fibroblasts and their transition into myofibroblasts; the transformation of collagen constituents (from type III collagen to type I collagen) facilitates the maturation of scar tissue^8^; and fibronectin and elastic fibres contribute to the remodelling of the dermal architecture.^9^, ^10^ Dysregulation of these meticulously orchestrated processes can result in aberrant deposition and disorganised alignment of the ECM, thereby exacerbating fibrosis and rigidity within scar tissues. Thus, these findings strongly emphasise the critical need to elucidate the key mechanisms modulating ECM deposition and organisation.

N6‐methyladenosine (m^6^A) is the most abundant internal modification identified in eukaryotic messenger RNAs (mRNAs) and has a major influence on numerous pathological and physiological conditions. Mechanistically, m^6^A modification is assembled by ‘writers’ methyltransferase‐like 3 (METTL3), methyltransferase‐like 14 (METTL14), METTL and Wilms’ tumour 1‐associated protein; removed by the ‘erasers’ fat mass and obesity‐associated protein (FTO) and ALKB family member 5 protein (ALKBH5)^11^; and recognised by the ‘readers’ YTH (YT521‐B homology) domain‐containing proteins (YTHDF1/2/3), heterogeneous nuclear ribonucleoprotein (HNRNP) and IGF2 mRNA‐binding proteins (IGF2BP).^12^ This dynamic post‐transcriptional modification governs the fate of mRNAs in multiple dimensions of RNA metabolism, including splicing, stability, translation, cytoplasmic transportation, and microRNA processing. As a result, m^6^A modifications garnered growing attention for their role in the pathogenesis of human disease.

Recently, emerging studies have revealed dysregulation of m^6^A methylation in various fibrotic diseases. For example, decreased *METTL3* in hepatic stellate cells (HSCs) was shown to lead to reduced m^6^A modification on *Lats2*, resulting in suppressed HSC activation and alleviation of liver fibrosis.^13^ Moreover, 1‐nitropyrene promoted ALKBH5 SUMOylation and subsequent proteasomal degradation; consequently, increasing the m^6^A modification of *FBXW7* contributed to pulmonary fibrosis by regulating alveolar cell senescence.^14^ Although ALKBH5 is an important regulator of re‐epithelialisation during cutaneous wound healing,^15^ an in‐depth understanding of the m^6^A landscape and functional candidates involved in dermal pathological fibrosis is lacking.

In this study, we identified a significant increase in m^6^A modification within HTS lesions via bioinformatics screening and correlation analysis that was attributed to the downregulation of ALKBH5 in fibroblasts. Our results demonstrated that the suppression of ALKBH5 resulted in scar hyperplasia characterised by excessive and disorganised ECM deposition, both in vitro and in vivo. By combining RNA sequencing (RNA‐seq), methylated RNA immunoprecipitation sequencing (MeRIP‐seq), and RNA‐binding protein immunoprecipitation (RIP‐qPCR), we identified key ECM components (including COL3A1, COL1A1 and ELN), as downstream targets of ALKBH5. Mechanistically, the downregulation of ALKBH5 increased the m^6^A modification of *COL3A1*, *COL1A1* and *ELN*. Subsequently, YTHDF1 recognised these m^6^A sites and stabilised their corresponding mRNAs, contributing to enhanced RNA expression of these ECM components. Overall, our data revealed a novel epigenetic mechanism of dysregulated ECM remodelling in HTS, which indicated direct modulation of key ECM components by ALKBH5 in a YTHDF1‐m^6^A‐dependent manner.

## 2 RESULTS

### 2.1 Insufficient ALKBH5 expression leads to enhanced global m^6^A modification in HTSs

**FIGURE 1:**
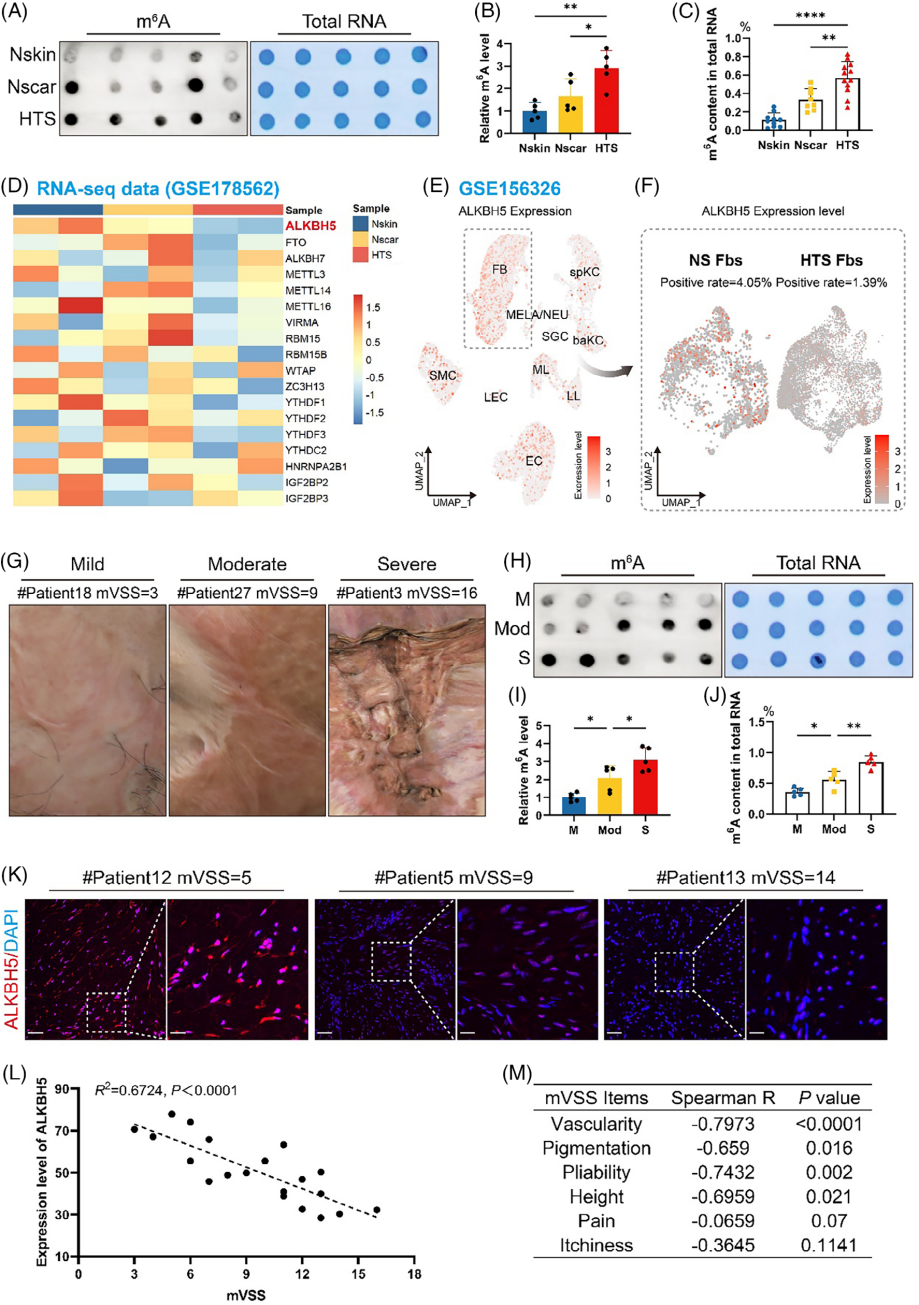
Downregulation of ALKBH5 in fibroblasts activates N6‐methyladenosine (m^6^A) modification in hypertrophic scar (HTS) lesions. (A and B) Global m^6^A modification levels of mRNA extracted from normal skin (Nskin), normal scar (Nscar) and HTS tissues were measured by m^6^A dot blot assays. Total RNA volume was determined by methylene blue staining, which served as a loading control. The images are representative of experimental triplicates. The data are presented as the mean ± standard deviation (SD). ^*^ *p *< .05, ^**^ *p *< .01. (C) An m^6^A RNA methylation assay revealed the m^6^A content in Nskin, Nscar and HTS tissues. For Nskin, *n* = 10; for Nscar, *n* = 8; for HTS, *n* = 12. ^****^ *p* < .0001, ^**^ *p* < .01. (D) Heatmap of m^6^A modifier gene expression in Nskin, Nscar and HTS according to the RNA‐seq data (GEO: GSE178562). (E) Feature plot depicting the expression distribution of *ALKBH5* in cells derived from normal skin and HTS tissues after the integration of all samples. The expression levels for each cell are colour‐coded and overlaid onto the uniform manifold approximation and projection (UMAP) plot; the dashed box indicates fibroblasts. Fibroblasts (Fbs), smooth muscle cells (SMCs), spinous keratinocytes (spKCs), basal keratinocytes (baKCs), endothelial cells (ECs), lymphatic endothelial cells (LECs), lymphoid leukocytes (LLs), myeloid leukocytes (MLs), melanocytes and neural cells (MELAs/NEUs) and sweat gland cells (SGCs). Single‐cell data were acquired from GSE156326. (F) Feature plots of the distribution of *ALKBH5* expression in fibroblasts, split by tissue. Colour intensity represents the expression levels of ALKBH5 in Nskin and HTS Fbs. The positive rates of ALKBH5 in Nskin and HTS Fbs are 4.05% and 1.39%, respectively. (G) Representative clinical photographs showing a cohort of patients with diverse clinical stages determined by the modified Vancouver Scar Scale (mVSS), ranging from mild to severe. From left to right, the panels are: # patient 18, mVSS = 3; # patient 27, mVSS = 9; # patient 3, mVSS = 16. (H and I) m^6^A dot blot showing m^6^A levels in HTS lesions at different clinical stages. The data are presented as the means ± SDs of triplicate experiments. ^*^ *p *< .05. M, mild; Mod, moderate; S, severe. (J) An m^6^A RNA methylation assay revealed the m^6^A content in different clinical stages. For each group, *n* = 5. ^*^ *p* < .05, ^**^ *p* < .01. (K) ALKBH5 expression levels in the dermis of mild, moderate and severe HTSs were visualised by immunofluorescence. Scale bar: upper panel, 100 µm; lower panel, 20 µm. (L) Pearson's *R* correlation plot of the expression level of ALKBH5 and the mVSS score (*n* = 20). (M) Correlation of ALKBH5 and individual items of the mVSS.

It has been well‐established that ECM deposition serves as the initial trigger for HTS pathogenesis,^16^ and we first determined the m^6^A landscape of HTSs as an ECM‐related disease model. To assess the global m^6^A methylation level of HTSs, we performed an anti‐m^6^A dot blot assay and an m^6^A RNA methylation assay. Our findings revealed an increased m^6^A methylation level in HTS lesions compared to that in normal skin and scar tissues (Figure 1A–C), consistent with previous m^6^A sequencing data (Figure S1A). We subsequently analysed genome‐wide transcriptomic data (Gene Expression Omnibus [GEO]: GSE178562) and screened for dysregulated RNA modifiers in HTSs. Among all the m^6^A‐related regulators, ALKBH5 exhibited a significant trend of downregulation in the HTSs (Figure 1D). We also investigated the expression levels of additional RNA modifiers involved in N1‐methyladenosine (m^1^A), N5‐methylcytosine (m^5^C) and N7‐methylguanosine (m^7^G) modification (Figure S2A), which were not significantly altered. Taken together, these bioinformatic data potentially underscore the importance of ALKBH5 during ECM deposition.

To further elucidate the modulation pattern of ALKBH5 in HTS at single‐cell resolution, we conducted single‐cell transcriptome analysis using public single‐cell RNA sequencing (scRNA‐seq) data obtained from human HTSs and healthy skin tissues (GEO: GSE156326). The feature plot illustrating the expression distribution of ALKBH5 in normal skin and HTS tissues was superimposed onto the unsupervised uniform manifold approximation and projection (UMAP) clustering plot (Figure 1E), revealing an enrichment of ALKBH5 in fibroblasts. Given that fibroblasts exhibit the most prominent alterations in the pathogenesis of HTSs, characterised by a distinct fibrotic signature, we specifically assessed the expression levels of ALKBH5 in fibroblasts derived from HTSs and normal skin (Figure 1F). Notably, we observed a substantial decrease in ALKBH5 expression in fibroblasts derived from HTSs, marked by a reduction in the proportion of ALKBH5‐positive fibroblasts. These multi‐omic analyses indicated that the m^6^A modification level was elevated in HTSs due to the downregulation of the m^6^A ‘eraser’ ALKBH5 in fibroblasts.

Moreover, we recruited a clinical cohort of 30 patients with HTS (Figure 1G; clinical information is displayed in Table S1) and assessed their clinical severity using the modified Vancouver Scar Scale (mVSS)^17^ (Table S2). Notably, the activation of m^6^A modification was correlated with an advanced stage of HTSs (Figure 1H–J). Then, we evaluated the expression level of ALKBH5 in HTS lesions across different clinical stages by immunofluorescence assays (Figure 1K). The results indicated that the expression of ALKBH5 was negatively correlated with the severity of the HTS, as demonstrated by the mVSS (*R* ^2^ = .6724, *p *< .0001, Pearson's *R* correlation coefficient) (Figure 1L). Spearman's correlation analysis revealed that scar vascularity and pliability are the clinical features most closely related to the expression level of ALKBH5 (Figure 1M). We found that the expression of FTO, another m^6^A ‘eraser’, was also decreased in HTSs (Figure 1D), but it did not display a similar correlation with clinical severity (*R* ^2^ = .02265, *p* > .05, Pearson's *R* correlation coefficient) (Figure S3A,B). Collectively, our observations suggested that ALKBH5‐mediated m^6^A modification may play a pivotal role in the epigenetic pathogenesis of HTSs.

### 2.2 Silencing Alkbh5 facilitates dermal remodelling of ECM components in HTSs

**FIGURE 2:**
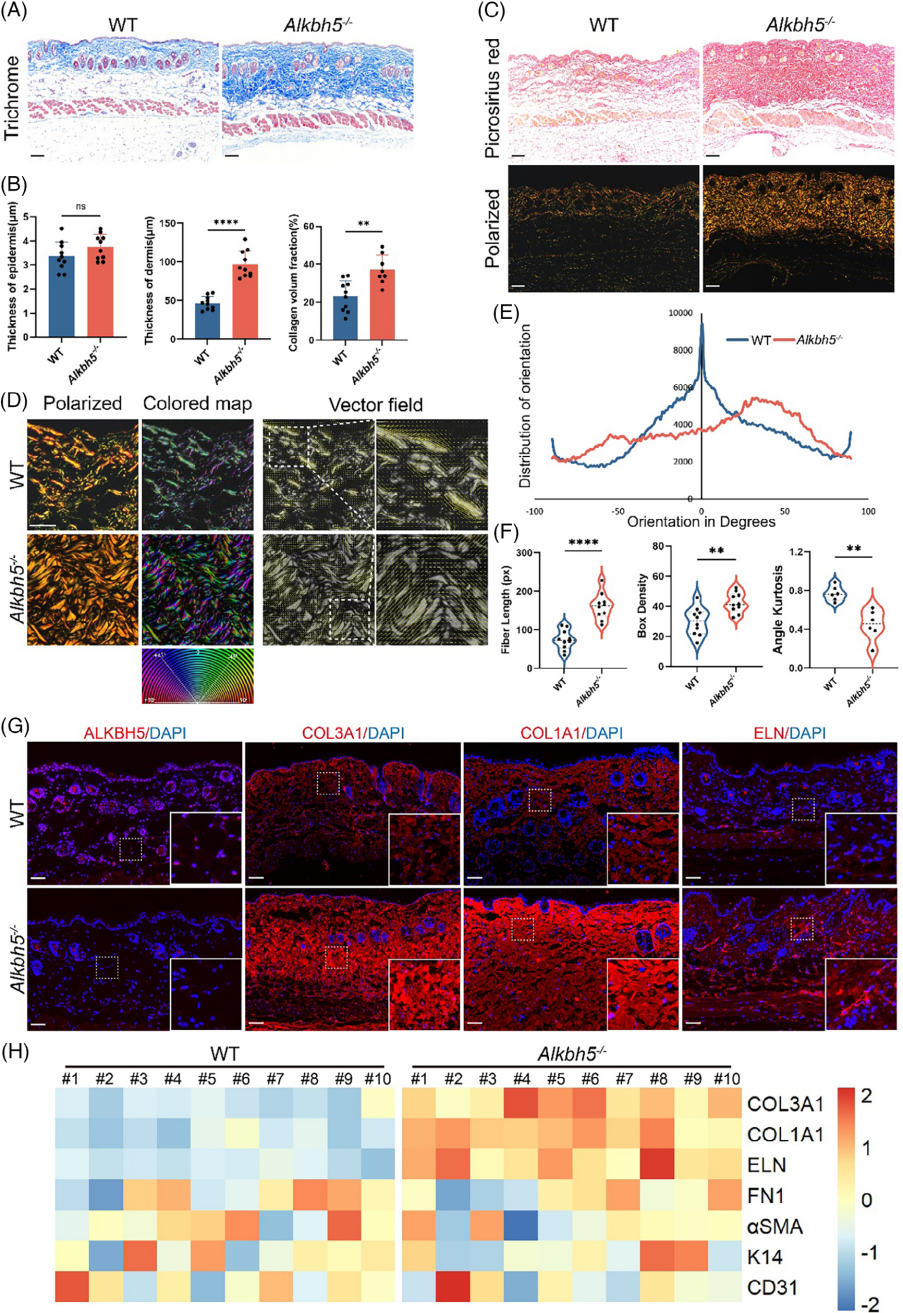
Loss of *Alkbh5* exacerbates disarranged dermal extracellular matrix (ECM) deposition. (A) Masson's trichrome staining illustrates the structural features and collagen deposition in the skin tissues of wild‐type (WT) and *Alkbh5^−/−^* mice (*n* = 10 biologically independent animals). Scale bar: 200 µm. (B) Statistical analysis of epidermal and dermal thickness and the collagen volume fraction (CVF). The data are presented as the mean ± standard deviation (SD). ns, not significant, ^**^ *p *< .01, ^****^ *p *< .0001. (C) Images of picrosirius red‐stained sections of WT and *Alkbh5^−/−^* mice under original light and polarised light. Scale bar: 200 µm. (D) Fibre orientation analysis was conducted on picrosirius red‐stained images under polarised light by Orientation J software. The coloured map and vector field panel visualise the local orientation, coherency and density of the fibres. Scale bar: 100 µm. (E) Quantitative analysis of the distribution of orientations by Orientation J software. (F) Quantification of the different collagen fibre network characteristics, fibre length, box density and angle kurtosis using the software algorithm CT‐FIRE. The data are presented as the mean ± SD. ^**^ *p *< .01, ^****^ *p *< .0001. (G) The expression levels of ALKBH5 and ECM components (COL3A1, COL1A1 and ELN) in the skin tissues of WT and *Alkbh5^−/−^* mice were visualised by immunofluorescence. Scale bar: 200 µm. (H) Heatmap depicting the expression levels of ECM components (COL3A1, COL1A1, ELN, FN1 and αSMA), epidermal constituents (K14) and vascular elements (CD31) in the skin tissues of WT and *Alkbh5^−/−^* mice.

To dissect the unique role of ALKBH5 in scar formation, we constructed *Alkbh5*‐knockout (*Alkbh5^−/−^*) mouse model with Exon1 deletion on *Alkbh5*.^15^ Subsequently, we observed the morphological features of the skin in the *Alkbh5^−/−^* mice to elucidate the specific role of ALKBH5 in the pathophysiological process of the skin. Histologically, compared with those of their WT littermates, the dermal thickness of the *Alkbh5^−/−^* mice significantly increased, and the levels of ECM components increased, while the thickness of the epidermal layer remained unchanged (Figure 2A,B). Additionally, a detailed quantitative assessment of the dermis was performed to analyse the fibre architecture using the software algorithms CT‐FIRE and Orientation J (Figure 2C,D). The fibre distribution in the dermis of the *Alkbh5^−/−^* mice was scattered, and these mice exhibited distinct collagen properties compared to those of the WT mice, characterised by elongated fibres, increased collagen density, and disrupted angle kurtosis (Figure 2E,F).

ECM volume was substantially increased in the dermis of the *Alkbh5^−/−^* mice, as shown by Masson's trichrome, picrosirius red and Victoria blue staining (Figures 2A,C and S4A,B). Hence, we investigated the deposition of ECM components in the dermis. Immunofluorescence assays revealed elevated expression levels of ECM components in the dermis of the *Alkbh5^−/−^* mice compared to those in the dermis of the WT mice, with collagen fibres and elastic fibres showing significant upregulation, while the expression levels of αSMA and FN1 remained consistent (Figure 2G,H). In addition, we measured the primary cellular components in the skin, including fibroblasts, keratinocytes and vascular endothelial cells; however, no significant differences in these cell types were observed between the *Alkbh5^−/−^* and WT mice (Figure S5A–C). We also investigated the immune land scape in the skin tissue of Alkbh5^‒/‒^ mice, and observed a downregulation of IL‐33 in ALKBH5‐deficient skin (Figure S6A–C). It suggested a complex interplay, indicating that ALKBH5's potential role in regulating the inflammatory response and vascular permeability. Taken together, these findings indicated that ALKBH5 is essentially involved in the process of ECM deposition and organisation in the dermis.

### 2.3 ALKBH5 ablation results in scar hyperplasia

**FIGURE 3:**
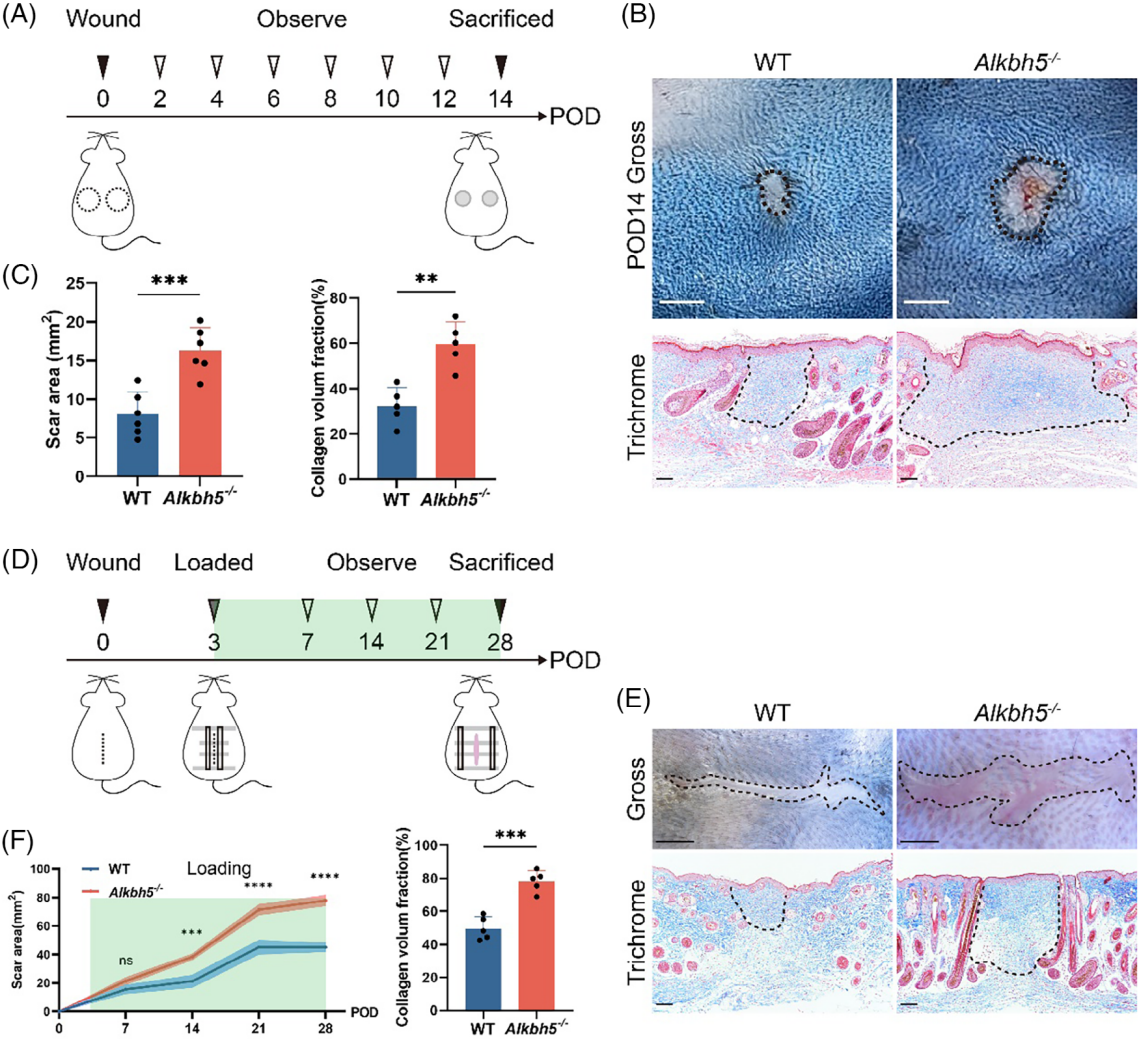
Depletion of *Alkbh5* intensifies scar hyperplasia in in vivo scar models. (A) Study design of the wound scarring model (*n* = 6 biologically independent animals). (B) Representative images of gross appearance (top, scale bar: 2 mm) at postoperative day 14 (POD14) and Masson's trichrome‐stained sections (bottom, scale bar: 200 µm) of the scars. The black dashed circle delineates the original 8 mm wide wound (top, scale bar: 3 mm), and the dashed lines outline the scar area (bottom, scale bar: 200 µm). (C) Quantification of the scar area at POD14 (left panel) and the collagen volume fraction (CVF) of the scars (right panel). The data are presented as the mean ± standard deviation (SD). ^**^ *p *< .01, ^***^ *p *< .001. (D) Study design of the mechanical stretch‐induced hypertrophic scar (HTS) model (*n* = 5 biologically independent animals). (E) Representative images of gross appearance (top) at POD28 and Masson's trichrome‐stained sections (bottom) of scars. The black dashed lines outline the scar area. (F) Quantitative analysis of the scar area at each observation site (left panel) and the CVF of the scars (right panel). The data are presented as the mean ± SD. ns, not significant, ^**^ *p *< .01, ^***^ *p *< .001, ^****^ *p *< .0001.

To delineate the functional role of ALKBH5 in scar formation, we subjected WT and *Alkbh5^−/−^* mice to several well‐established scar models. Scarring is an intricately coordinated, long‐lasting procedure that typically commences approximately 2 weeks post‐wounding.^6^ Accordingly, we harvested early‐stage scar tissue after full‐thickness skin wounds were created on the backs of the mice on postoperative day 14 (POD14) (Figure 3A). The results showed a notable extension of the gross scar area in the *Alkbh5^−/−^* mice, concomitant with increased ECM deposition within the dermis (Figure 3B,C). Quantitative analysis further revealed differences in the arrangement of the collagen fibres in the *Alkbh5^−/−^* mice compared to those in the WT mice, characterised by denser and more disorganised collagen fibres (Figure S7A–C). Interestingly, no significant dynamic changes in ALKBH5 expression were observed in the dermis throughout the process of scar formation (Figure S8A,B).

We then generated a mechanical stretch‐induced HTS model^18^ in which mechanical stretch was applied from POD3 until scar maturation (Figure 3D). Gross morphology and histologic trichrome staining indicated significant scar hyperplasia in the *Alkbh5^−/−^* mice (Figure 3E,F). In particular, abnormal ECM assembly was observed in the stretch‐derived HTSs of the *Alkbh5^−/−^* mice, demonstrating rigid, thickened and dysregulated collagen fibres (Figure S9A–C). Additionally, we established a classic bleomycin‐induced fibrotic scar model to validate the effects of ALKBH5 on the pathogenesis of skin fibrosis; the results were similar to those in the other two scar models (Figure S10A–C).

Collectively, these observations revealed that ALKBH5 serves as an anti‐scarring factor, modulating the deposition and organisation of dermal ECM during the initial phase of scar development. A lack of ALKBH5 results in scar hyperplasia and fibrosis, and this impact is not limited to specific scar models.

### 2.4 Downregulation of ALKBH5 stimulates the synthesis of ECM components in vitro

**FIGURE 4:**
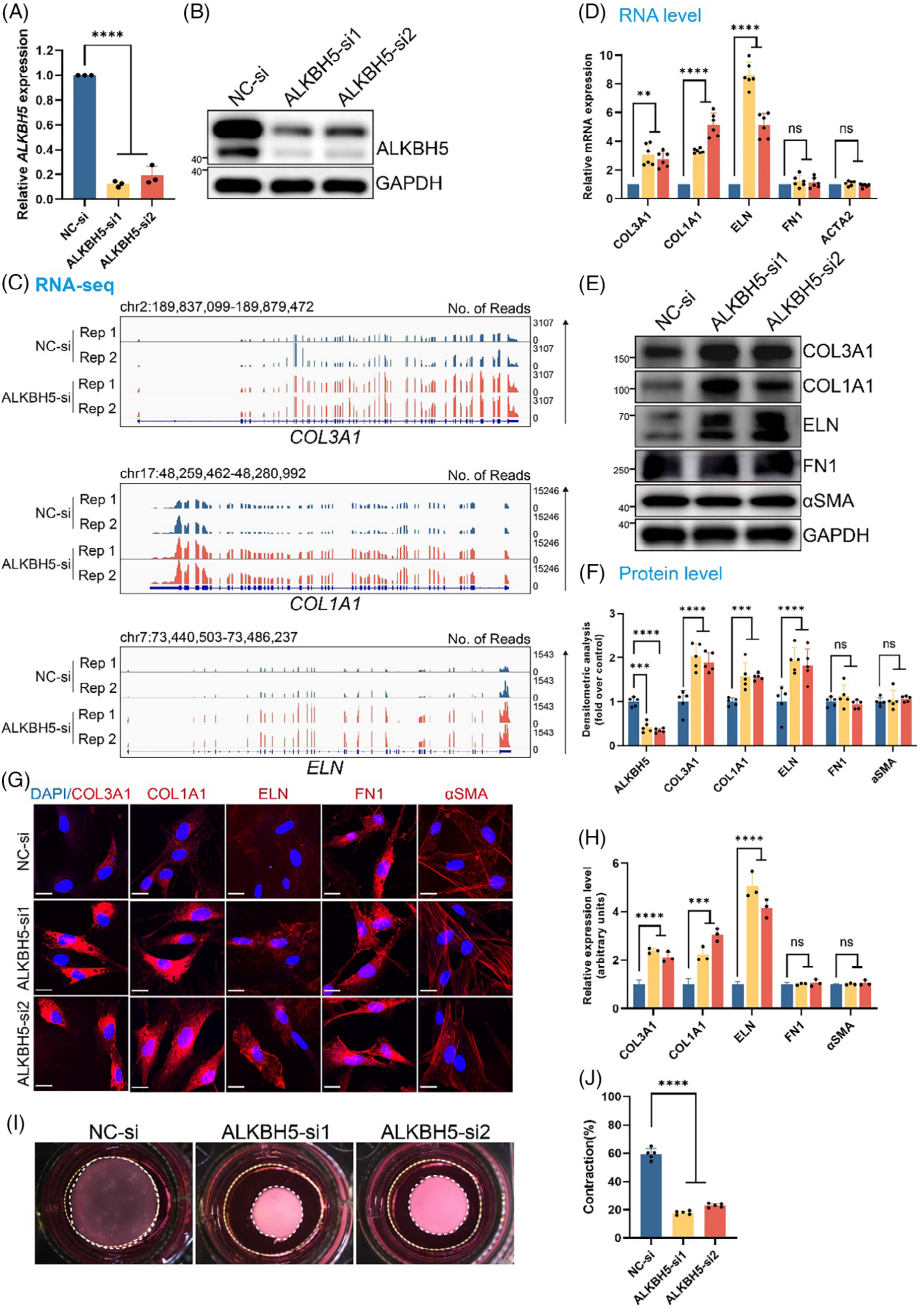
Inhibition of ALKBH5 facilitates extracellular matrix (ECM) synthesis in fibroblasts in vitro. (A and B) Decreased ALKBH5 expression was confirmed in human dermal fibroblasts (HDFs) after siRNA transfection by qRT‐PCR (A) and Western blot (WB) (B). The experiments were performed in triplicate, and the data are presented as the mean ± standard deviation (SD). ^****^ *p *< .0001. (C) Integrative Genomics Viewer (IGV) tracks of ECM components (*COL3A1*, *COL1A1* and *ELN*) according to the RNA‐seq analysis of ALKBH5 knockdown or control HDFs. The experiments were performed in duplicate. (D) The expression levels of major ECM components (*COL3A1*, *COL1A1*, *ELN*, *FN1* and *ACTA2*) in *ALKBH5* knockdown and control HDFs were measured by qRT‐PCR. The data are presented as the mean ± SD. ns, not significant, ^**^ *p *< .01, ^****^ *p *< .0001. (E and F) The protein levels of major ECM components (COL3A1, COL1A1, ELN, FN1 and αSMA) in *ALKBH5* knockdown and control HDFs. Quantitative data are presented as the mean ± SD. ns, not significant, ^***^ *p *< .001, ^****^ *p *< .0001. (G and H) Immunofluorescence showing the protein expression of major ECM components (COL3A1, COL1A1, ELN, FN1 and αSMA) in HDFs. Scale bar: 25 µm. Quantitative data are presented as the mean ± SD. ns, not significant, ^***^ *p *< .001, ^****^ *p *< .0001. (I and J) Images and quantification of collagen gel contraction in *ALKBH5* knockdown and control HDFs. The dashed lines indicate the area of the collagen gel. The data are presented as the mean ± SD. ^****^ *p *< .0001.

Although we demonstrated the antifibrotic function of ALKBH5 in the in vivo scar formation process, its impact on the biological behaviour of dermal fibroblasts remains unclear. Consequently, we silenced *ALKBH5* in human dermal fibroblasts (HDFs) derived from healthy skin by transfecting two individual small interfering RNAs (siRNAs). As a result, the expression level of ALKBH5 decreased by approximately 80% compared to that of the normal control (Figure 4A,B). We then conducted a genome‐wide transcriptome analysis to delineate the transcriptomic landscape of the *ALKBH5*‐silenced HDFs (GEO accession number: GSE264515), revealing a distinct expression pattern, with 2598 upregulated and 2335 downregulated transcripts in the *ALKBH5*‐silenced group (Figure S11A). Notably, Gene Ontology (GO) analysis and Gene Set Enrichment Analysis revealed substantial enrichment of ECM‐related pathways in the *ALKBH5*‐deprived HDFs, including genes related to ECM organisation, the cytoskeleton, and elastic fibres (Figure S11B,C).

We screened the expression levels of several dermal ECM components previously reported in HTS studies.^19^ Specifically, *COL3A1*, *COL1A1* and *ELN* were upregulated upon *ALKBH5* knockdown (Figure 4C), while *FN1*, *ACTA2*, *COL5A2*, *COL4A5*, *COL7A1* and *COL11A1* remained unchanged (Figure S12A). Accordingly, the elevation of *COL3A1*, *COL1A1* and *ELN* was also corroborated using qRT‐PCR, Western blot (WB) and immunofluorescence staining assays (Figure 4D–H).

Moreover, the biological functions of ALKBH5 in HDFs were subsequently investigated. Depletion of *ALKBH5* resulted in negligible changes in cell proliferation, cell cycle and apoptosis, as demonstrated by Cell‐Counting‐Kit‐8 (CCK‐8) (Figure S13A), 5‐ethynyl‐2′‐deoxyuridine (EdU) (Figure S13B,C) and flow cytometry assays (Figure S13D–G), respectively. The migratory velocity and invasive capacity also remained unaffected by *ALKBH5* deficiency, as confirmed by wound healing (Figure S14A,B) and Transwell assays (Figure S15A,B). Additionally, using a contraction assay, we observed that *ALKBH5*‐deficient HDFs exhibited an aggressive ability to remodel the surrounding ECM environment (Figure 4I,J). These results indicated that ALKBH5 specifically regulates the synthesis of ECM components in dermal fibroblasts rather than influencing other biological behaviours, such as proliferation, apoptosis or cellular migration.

### 2.5 COL3A1, COL1A1 and ELN are downstream candidates of ALKBH5

**FIGURE 5:**
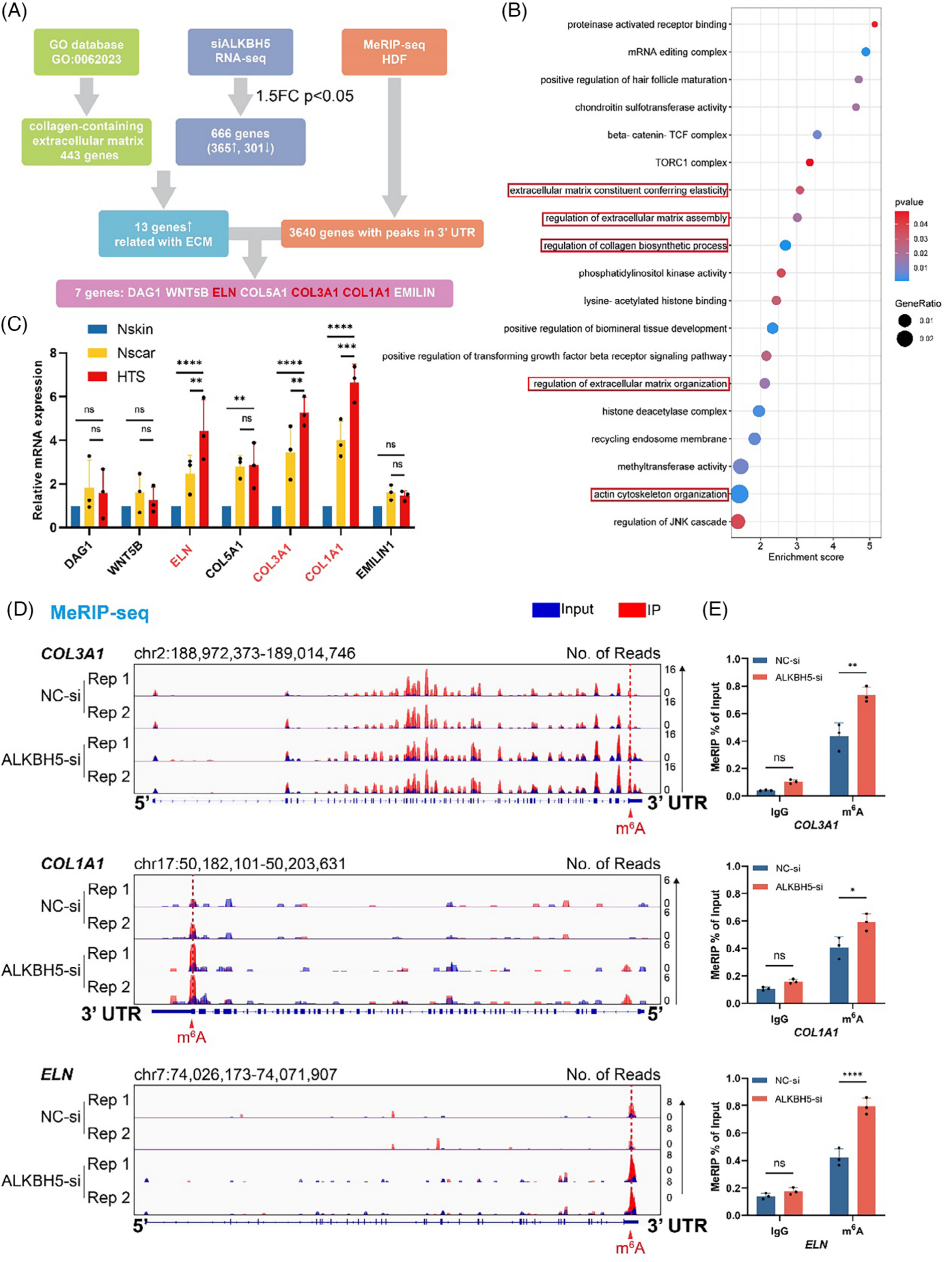
Extracellular matrix (ECM) components COL3A1, COL1A1 and ELN serve as candidates downstream of ALKBH5. (A) Bioinformatics analysis of N6‐methyladenosine (m^6^A) modification downstream targets. RNA‐seq revealed 365 upregulated genes in *ALKBH5* knockdown human dermal fibroblasts (HDFs), 13 of which were related to ECM components according to the Gene Ontology (GO) database (GO: 0062023) annotated as ‘collagen‐containing extracellular matrix’. Methylated RNA immunoprecipitation sequencing (MeRIP‐seq) identified 3640 genes with specific m^6^A peaks in the 3′ UTR. Seven genes (*DAG1*, *WNT5B*, *ELN*, *COL5A1*, *COL3A1*, *COL1A1* and *EMILIN*) were identified as potential candidates for ALKBH5. (B) GO enrichment map of genes with m^6^A peaks in the 3′ UTR. (C) The expression levels of the seven candidate genes in normal skin, normal scar and hypertrophic scar (HTS) tissues measured by qRT‐PCR. The data are presented as the mean ± standard deviation (SD). ns, not significant, ^**^ *p *< .01, ^***^ *p *< .001, ^****^ *p *< .0001. (D) IGV tracks displaying the MeRIP‐seq read coverage of *COL3A1*, *COL1A1* and *ELN* in the control and ALKBH5 knockdown HDFs. (E) m^6^A‐RIP‐qPCR assays confirmed the m^6^A modification of the *COL3A1*, *COL1A1* and *ELN* transcripts. The experiments were performed in triplicate. The relative mRNA expression in the anti‐m^6^A antibody group was compared to that in the immunoglobulin G (IgG) group. The data are presented as the mean ± SD. ns, not significant, ^*^ *p *< .05, ^**^ *p *< .01, ^****^ *p *< .0001.

To systematically elucidate the regulatory role of the ALKBH5/m^6^A‐mediated mechanism in excessive dermal ECM deposition and identify its downstream candidates, we mapped m^6^A modification sites in HDFs with or without *ALKBH5* knockdown by MeRIP‐seq utilising two independent biological replicates (GEO accession number: GSE264743). On average, 9791 and 11 389 m^6^A peaks were identified in the control and *ALKBH5*‐silenced HDFs, respectively, consistent with the recognised function of ALKBH5 as a classic m^6^A ‘eraser’ (Figure S16A). Consistent with previous studies, these peaks were enriched in the 3′ UTR, especially near the stop codons (Figure S16B,C), and were characterised by the canonical RRACH (R = G or A; H = A, C or U) motif (Figure S16D). In total, we identified 3640 genes that exhibited significant m^6^A modifications in the 3′ UTRs of their corresponding mRNAs across all tested replicates. GO analysis revealed prominent enrichment of these genes in terms of the regulation of ECM organisation and collagen biosynthetic processes, indicating a regulatory role of ALKBH5 in dermal ECM remodelling (Figure 5B).

Thirteen of 365 upregulated genes in the *ALKBH5* knockdown HDFs were found to be associated with ECM components, as indicated by GO database annotation of collagen‐containing ECM (GO:0062023). Next, through the integration of MeRIP‐seq data (GSE264743) with RNA‐seq data (GSE264515), we identified seven potential downstream targets characterised as upregulated ECM‐related genes in the *ALKBH5*‐silenced HDFs, with m^6^A peaks detected in the 3′ UTRs of their corresponding mRNAs (Figure 5A).

The expression levels of the seven target genes were assessed in the clinical samples. Notably, the expression of *COL3A1*, *COL1A1* and *ELN* was significantly greater in the HTS lesions than in both the normal skin and scar tissues (Figure 5C). Therefore, *COL3A1*, *COL1A1* and *ELN* were identified as crucial downstream candidates of ALKBH5 in the pathogenesis of HTSs. Our MeRIP‐seq data revealed statistically significant m^6^A peaks in the mRNA transcripts of *COL3A1*, *COL1A1* and *ELN* within the 3′ UTR, with particularly high enrichment observed in the *ALKBH5*‐silenced HDFs (Figure 5D). To confirm these results, we performed an RIP‐qPCR assay and observed abundant enrichment of m^6^A signals in *COL3A1*, *COL1A1* and *ELN* after silencing *ALKBH5* (Figure 5E). Taken together, ALKBH5 is likely to influence the deposition and organisation of ECM components in HTSs via specific modulation of m^6^A modifications in *COL3A1*, *COL1A1* and *ELN*.

### 2.6 YTHDF1 recognition of m^6^A increases the RNA stability of COL3A1, COL1A1 and ELN

**FIGURE 6:**
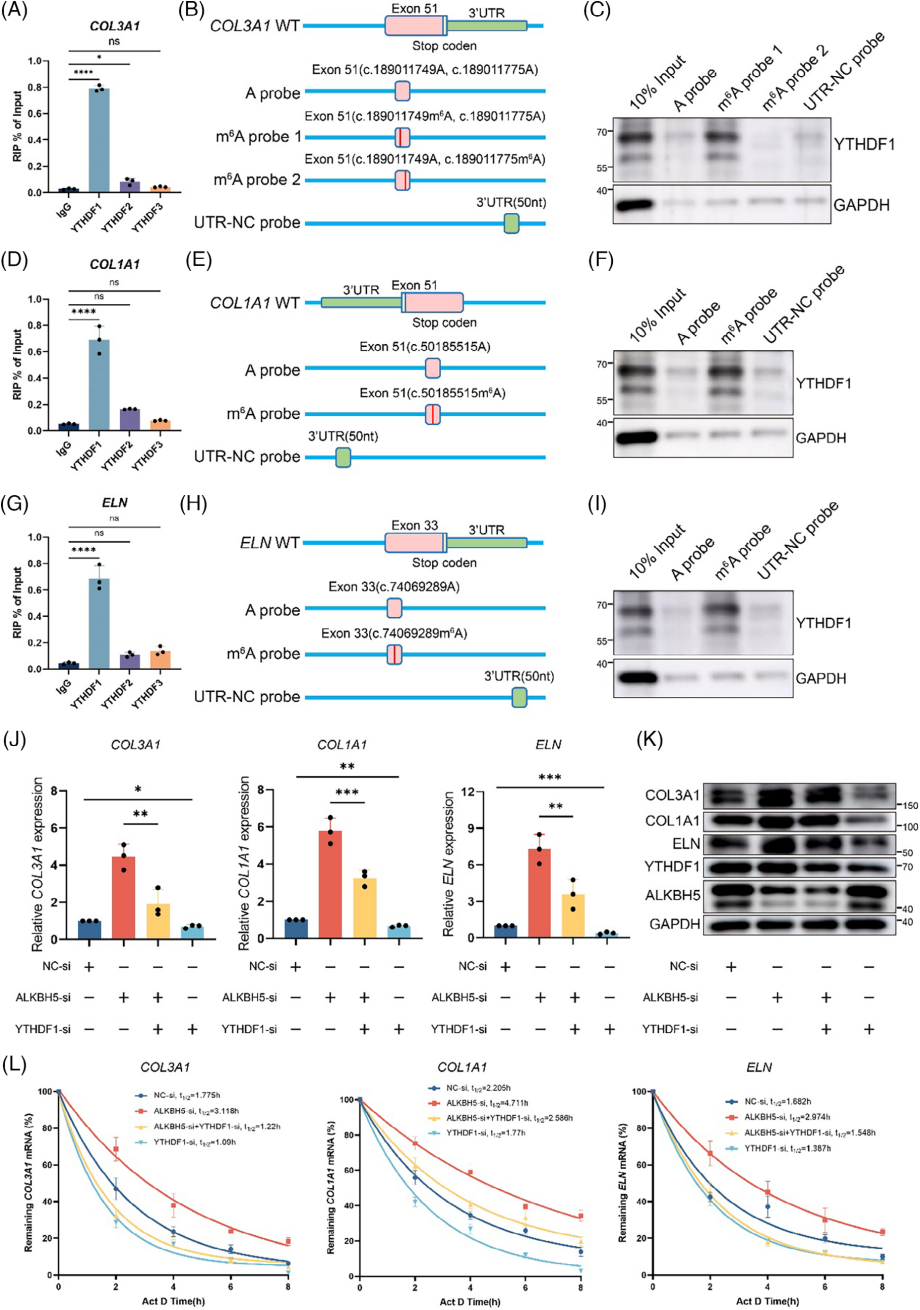
The recognition of COL3A1, COL1A1 and ELN N6‐methyladenosine (m^6^A) by YTHDF1 increases RNA stability. (A, D, G) RIP‐qPCR analysis revealed the enrichment of YTHDF1, YTHDF2 and YTHDF3 in the *COL3A1*, *COL1A1* and *ELN* transcripts. The data are presented as the mean ± standard deviation (SD). ns, not significant, ^*^ *p *< .05, ^****^ *p *< .0001. (B, E, H) Diagrams of the RNA probes used for RNA pull‐down assays. (C, F, I) RNA pulldown of endogenous YTHDF1 proteins from human dermal fibroblasts (HDFs) extracted using *COL3A1*, *COL1A1* or *ELN* RNA fragments with or without m^6^A modifications. Images are representative of three independent experiments. (J and K) The expression levels of COL3A1, COL1A1 and ELN in *ALKBH5* knockdown HDFs with or without YTHDF1 silencing were assessed by qRT‐PCR and Western blot (WB) analyses. The data are presented as the mean ± SD. ^*^ *p *< .05, ^**^ *p *< .01, ^***^ *p *< .001. (L) Lifetime of *COL3A1*, *COL1A1* and *ELN* mRNA levels were assessed in *ALKBH5* knockdown HDFs with or without YTHDF1 silencing.

Since YHTDF family proteins are responsible for recognising m^6^A modifications and orchestrating the fate of corresponding mRNA transcripts,^20^ we further investigated the role of these ‘readers’ in governing the regulation of *COL3A1*, *COL1A1* and *ELN*. Notably, RIP‐qPCR analysis revealed a robust interaction between YTHDF1 and *COL3A1*, *COL1A1* and *ELN*, whereas a minimal signal was found for YTHDF2 and 3 (Figure 6A,D,G).

Subsequently, utilising the MeRIP‐seq data, we identified ‘RRACH’ motifs within the transcripts of *COL3A1*, *COL1A1* and *ELN*, which allowed us to identify potential m^6^A modification sites in the corresponding mRNAs. By employing biotin‐labelled single‐stranded RNA probes, we confirmed the predominant binding sites of *COL3A1*, *COL1A1* and *ELN* (Figure 6B,E,H; Table S6). As observed in the RNA pull‐down assays, m^6^A modification of *COL3A1*, *COL1A1* and *ELN* increased their interactions with YTHDF1 (Figure 6C,F,I). In this context, we showed that YTHDF1 recognises m^6^A methylation of *COL3A1* at c.189011749, of *COL1A1* at c.50185515, and of *ELN* at c.74069289 within their mRNA transcripts. Consistently, silencing YTHDF1 inhibited the expression of *COL3A1*, *COL1A1* and *ELN*. Importantly, the upregulation of the three targets induced by *ALKBH5* silencing was reversed at both the mRNA and protein levels (Figure 6J,K), suggesting that YTHDF1 acts as a reader for methylated transcripts of *COL3A1*, *COL1A1* and *ELN* (Figure 6C,F,I).

YTHDF1 reportedly functions via a m^6^A‐mediated increase in mRNA stability in HeLa cells.^21^ To explore whether mRNA stability is affected, we conducted mRNA decay assays using the transcription inhibitor actinomycin D. Prolonged half‐lives of the *COL3A1*, *COL1A1* and *ELN* mRNAs were detected in *ALKBH5*‐silenced HDFs. Conversely, opposite results were found following YTHDF1 knockdown (Figure 6L).

### 2.7 Adeno‐associated virus‐capsulated overexpression of ALKBH5 therapeutically alleviates the formation of HTSs

**FIGURE 7:**
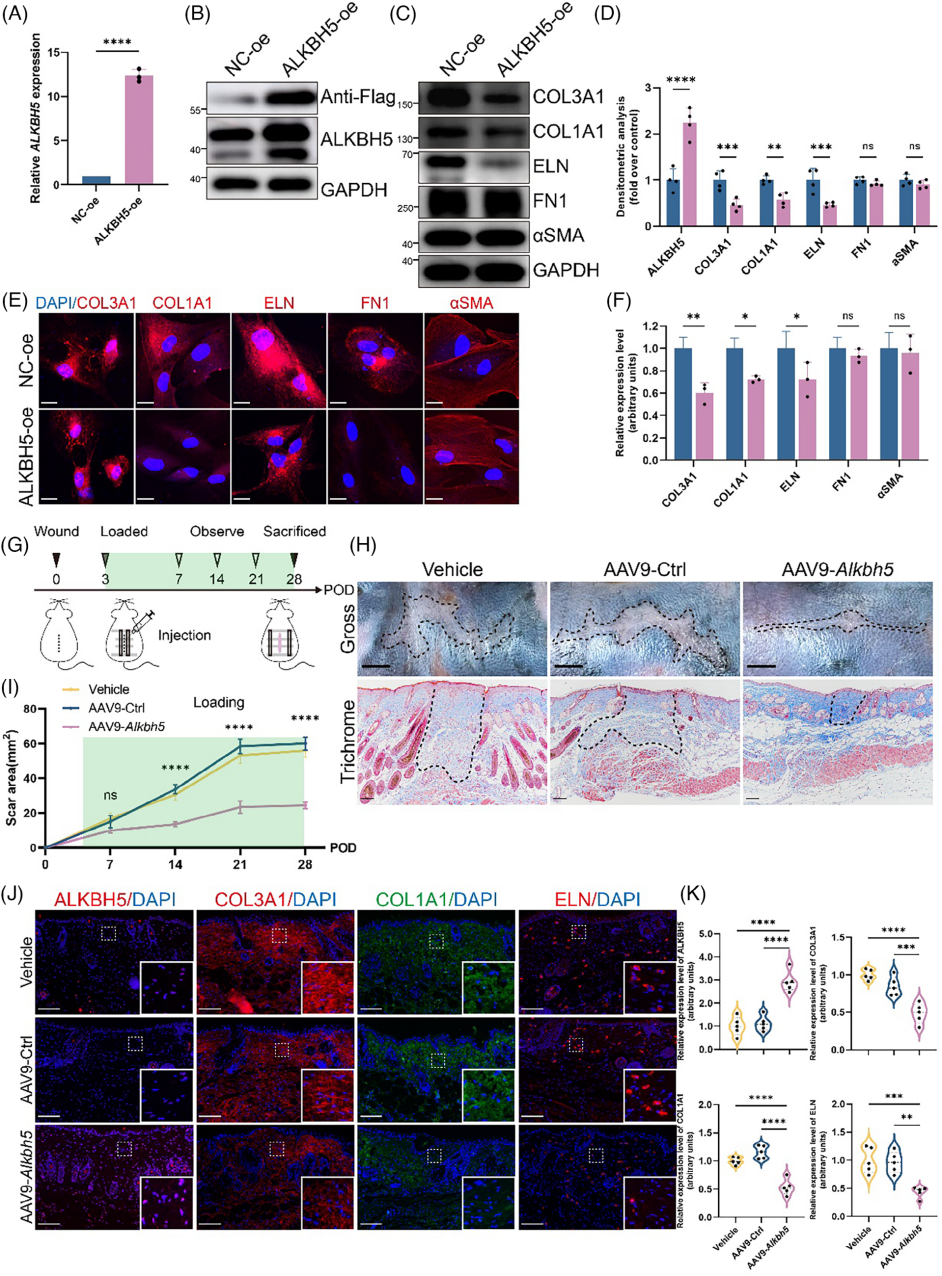
Exogenous *ALKBH5* overexpression attenuates extracellular matrix (ECM) deposition and scar formation. (A and B) Elevated ALKBH5 expression was confirmed in HTS‐derived fibroblasts (HSFs) after exogenous overexpression of *ALKBH5* by qRT‐PCR (A) and Western blot (WB) analyses (B). The data are presented as the mean ± standard deviation (SD). ^****^ *p *< .0001. (C and D) The protein levels of major ECM components (COL3A1, COL1A1, ELN, FN1 and αSMA) in *ALKBH5*‐overexpressed or control HSFs. The data are presented as the mean ± SD. ns, not significant, ^**^ *p *< .01, ^***^ *p *< .001, ^****^ *p *< .0001. (E and F) Immunofluorescence showing the protein expression of major ECM components (COL3A1, COL1A1, ELN, FN1 and αSMA) in *ALKBH5*‐overexpressed or control HSFs. Scale bar: 25 µm. The data are presented as the mean ± SD. ns, not significant, ^*^ *p *< .05, ^**^ *p *< .01. (G) Study design of the mechanical stretch‐induced hypertrophic scar (HTS) model. The intradermal injections of adeno‐associated virus (AAV) vectors or .9% saline were performed at postoperative day 3 (POD3), 4 and 5 (*n* = 5 biologically independent animals). (H) Representative images of gross appearance (top, scale bar: 2 mm) at POD28 and Masson's trichrome‐stained sections (bottom, scale bar: 200 µm) of the scars. The black dashed lines outline the scar area. (I) Quantitative analysis of the scar area at each observation site. The data are presented as the mean ± SD. ns, not significant, ^****^ *p *< .0001. (J and K) Immunofluorescence confirmed the increase in ALKBH5 by the AAV9‐*Alkbh5* vector and visualised the expression of COL3A1, COL1A1 and ELN. The signal density of arbitrary units was measured for quantitative analysis. Scale bar: 100 µm. The data are presented as the mean ± SD. ^**^ *p *< .01, ^***^ *p *< .001, ^****^ *p *< .0001.

To explore the therapeutic potential of exogenous supplementation with ALKBH5 in mitigating pathological ECM deposition, we induced *ALKBH5* overexpression in human HTS‐derived fibroblasts (HSFs) (Figure 7A,B). As expected, increased ALKBH5 expression specifically suppressed COL3A1, COL1A1 and ELN expression (Figures S18A and 7C,D). Immunofluorescence staining of HSFs further revealed that COL3A1, COL1A1 and ELN were downregulated in response to *ALKBH5* overexpression (Figure 7E,F). Additionally, *ALKBH5* overexpression had no discernible effect on the proliferation or apoptosis of HSFs (Figure S17A–G). Importantly, the pronounced contractile tendency of HSFs was inhibited by *ALKBH5* supplementation (Figure S18B,C). Collectively, these findings suggest that in vitro *ALKBH5* overexpression partially reversed the HTS phenotype. Besides, considering the feasibility of clinical applications, we also explored the therapeutic potential of small‐molecule drugs. Since no ALKBH5 agonists are currently available, we treated HSFs with STM2457, a small‐molecule inhibitor of METTL3,^22^ to eliminate the m6A modifications. However, our in vitro experiments indicated that STM2457 had negligible effect in reversing the fibrotic phenotype (Figure S19A–C).

**FIGURE 8:**
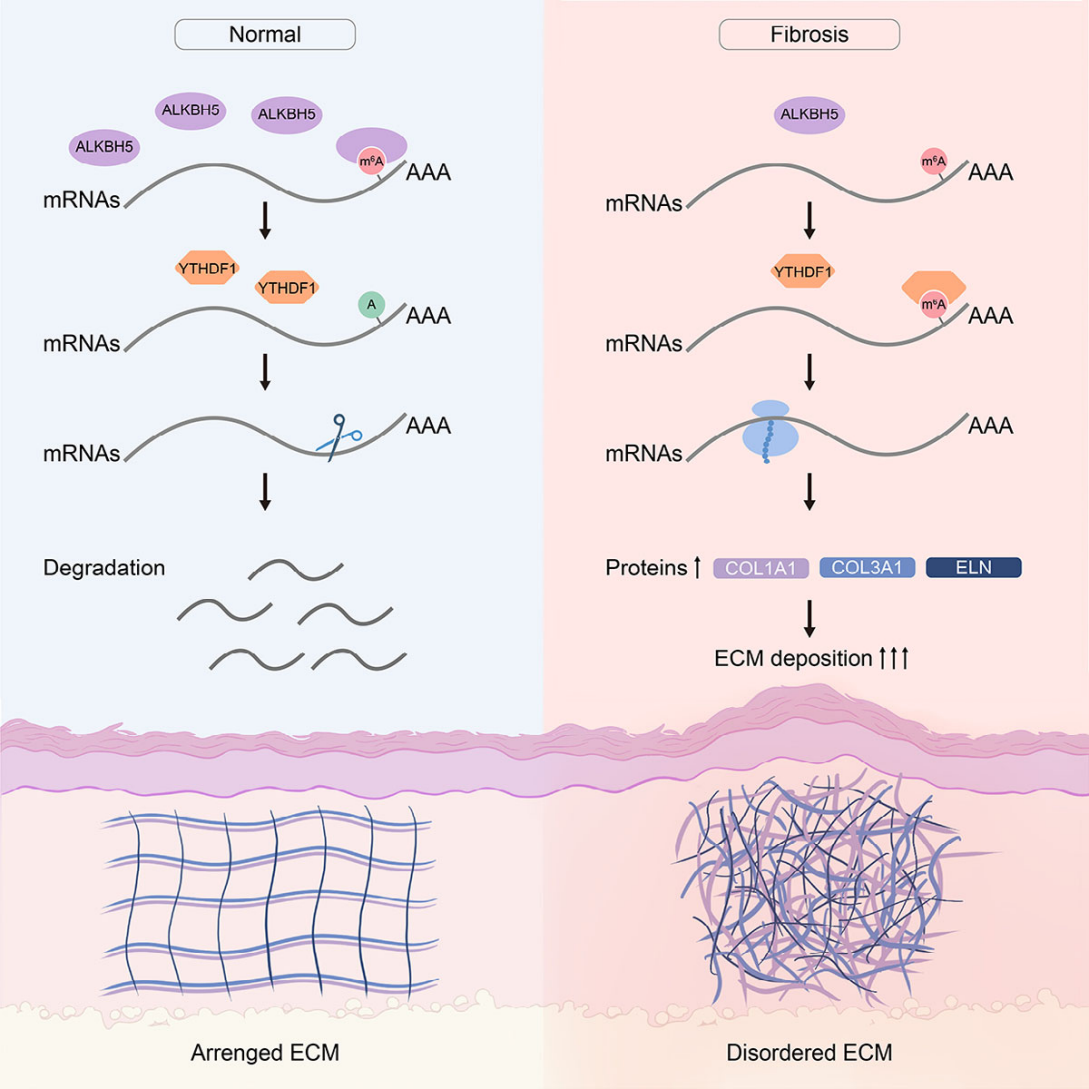
Schematic of excessive elevated extracellular matrix (ECM) component deposition in hypertrophic scars (HTSs) via an ALKBH5/N6‐methyladenosine (m^6^A)‐mediated mechanism. Downregulation of ALKBH5 promoted the m^6^A modification of downstream ECM targets (*COL3A1*, *COL1A1* and *ELN*) in HTS lesions. YTHDF1 subsequently recognises m^6^A modifications and stabilises the corresponding mRNAs. Therefore, the protein levels of COL3A1, COL1A1 and ELN increase, resulting in the excessive deposition of disordered ECM.

To further investigate the effect of ALKBH5 on scar formation in vivo, *Alkbh5* was administered to a stretch‐induced HTS in WT mice using an adeno‐associated virus (AAV) system (Figure 7G). The AAV vectors were intradermally administered for 3 days simultaneously with the application of mechanical stretch. Notably, AAV9‐*Alkbh5* treatment resulted in a significant reduction in scar area at each time point compared to those of both the AAV9‐Ctrl‐ and saline‐treated mice (Figure 7H,I). Histological analysis revealed a notable decrease in the cross‐sectional size and collagen density of scars in the AAV9‐*Alkbh5*‐treated mice (Figure 7H). In addition, immunofluorescence staining confirmed the overexpression of *Alkbh5* and demonstrated decreased expression of COL3A1, COL1A1 and ELN within the scar tissues of the AAV9‐*Alkbh5*‐treated mice (Figure 7J,K). Furthermore, the irregular collagen alignment was corrected by AAV9‐*Alkbh5* administration, resulting in a more flexible basket weave‐like collagen fibre network (Figure S20A–C). These findings collectively underscore the therapeutic potential of ALKBH5 as an antifibrotic agent for relieving HTSs Figure 8.

## 3 DISCUSSION

Fibrosis refers to a pathological state that is distinguished by dysregulation of ECM components, and this condition affects multiple organ systems, including but not limited to the heart, lungs, liver, kidneys and skin. Fibroblasts, which serve as the core of fibrotic pathogenesis, exhibit profound diversity, functional heterogeneity and plasticity, both within and between organs.^23^, ^24^ Accordingly, a more precise delineation of fibroblast phenotype within different disease settings is anticipated to facilitate the development of rational, highly targeted antifibrotic interventions.^1^ Recent investigations have revealed the pivotal regulatory role of m^6^A modification in both fibrogenic and antifibrotic processes.^25^, ^26^ Here, we discovered the antifibrotic effect of the m^6^A ‘eraser’ ALKBH5 on HTSs via an m^6^A‐mediated mechanism (Figure 8).

Through the combined analysis of RNA‐seq and scRNA‐seq data, we identified a decrease in ALKBH5 expression within fibroblasts in pathological skin fibrosis. Recently, several studies have also explored the role of ALKBH5 in organ fibrotic diseases such as pulmonary and renal fibrosis. Li et al. observed ALKBH5 SUMOylation and subsequent proteasomal degradation during 1‐nitropyrene‐induced pulmonary fibrosis.^14^ Similarly, another study on PM2.5 exposure‐induced pulmonary fibrosis reported a reduction in ALKBH5 levels, revealing that ALKBH5 deficiency exacerbated the fibrotic condition.^27^ Notably, promoted ECM deposition were also observed in lungs of ALKBH5‐deficient mice exposed to PM2.5, which aligns with our findings. However, a contrary trend was observed in ischaemia/reperfusion (I/R)‐induced acute kidney injury and fibrosis, where a decrease in ALKBH5 was found to protect against the I/R‐induced kidney injury and fibrosis by regulating the inflammatory process.^28^ These opposing results are not unexpected given the distinct pathological characteristics of organ fibrotic diseases caused by different aetiologies. Both skin fibrosis and pulmonary fibrosis are relatively long‐term fibrotic processes that primarily manifests as ECM deposition. While acute pathological process such as I/R‐induced kidney fibrosis is mainly driven by the regulation of inflammatory cell recruitment and inflammatory responses.

The dermal ECM is a critical structure with a dynamic and complex organisation and is mainly composed of abundant collagens and elastic fibres.^29^ For the first time, we revealed the direct regulatory effect of ALKBH5 on key ECM components, namely, COL3A1, COL1A1 and ELN. These downstream targets are recognised and stabilised by YTHDF1. As it was known that YTHDF1 can promote translation by interacting with ribosomes and translation‐initiating factors.^30^ YTHDF1 may increase the expression of target molecules through its ability to promote translation, but this issue requires further exploration.

In our findings, the suppression of ALKBH5 promoted the expression of COL3A1, COL1A1 and ELN, and these increased ECM components were subsequently secreted and deposited within the dermis, culminating in dermal fibrosis and scar hyperplasia. These phenomena were not confined to the stage of scar formation; rather, the immature scar and normal skin of *Alkbh5^−/−^* mice were also affected. Accordingly, these observations indicated that silencing *ALKBH5* hindered the capacity for skin regeneration and increased susceptibility to dermal fibrosis. Notably, early intervention in re‐epithelialised wounds to control HTS formation has been proposed and proven to be a feasible approach.^31^ Our previous research revealed impeded wound re‐epithelialisation in the absence of ALKBH5, which could be a detrimental factor contributing to scar hyperplasia.^15^

During ECM remodelling, there is a transition in the composition pattern of collagen fibres from type III to type I, which maintains the homeostasis of the dermis and mediates scar maturation.^32^ Therefore, increased crosslinking between collagen fibres and an inappropriate transition from immature type III collagen to mature type I collagen at the early stages of repair could cause fibrotic scarring.^33^ Notably, studies have reported a key role for COL3A1 in COL1A1 fibrillogenesis, consequently exacerbating the imbalance within collagen fibres.^34^, ^35^ In our study, a disproportionate increase in COL3A1 and COL1A1 was observed in the skin tissue of the *Alkbh5^−/−^* mice. Moreover, during the quantitative analysis of fibre orientation, we observed an elevated proportion of thick and long fibres following the silencing of *ALKBH5*. This phenomenon results in weakened anchoring of aberrant collagen fibres within the dermal scaffold and increased inter‐fibre crosslinking.^36^ Another notable finding of our research was that elastin was also a downstream target of ALKBH5. Previous research has implied that elastic fibres extend through the dermal layer and consist of a dense core of crosslinked elastin surrounded by aligned fibrillin microfibrils, thereby serving as the structural scaffold of the skin.^37^ Consequently, the impaired scaffolding role of elastin further mediates the structural disarray of collagen fibres, contributing to the disruption of fibre crosslinking. Overall, ALKBH5 exhibited a dual regulatory role, affecting both the quantity (total amount) and quality (arrangement structure) of the dermal ECM. The excessive deposition of disorganised ECM led to the stiffness of HTS lesions; in turn, the increased mechanical stimulation in the ECM microenvironment irritated fibroblasts.^38^, ^39^

Interestingly, despite the increase in ECM components, negligible fibroblast‐to‐myofibroblast transition occurred during the silencing of *ALKBH5* in vitro. Additionally, no significant difference in ALKBH5 expression levels was observed between α‐SMA‐positive (myofibroblasts) and α‐SMA‐negative fibroblasts (Figure S21A). Generally, the transient activation of fibroblasts into myofibroblasts is crucial for tissue repair, and their prolonged presence contributes to organ fibrosis.^40^ Transforming growth factor‐β1 (TGF‐β1) is one of the earliest studied and most potent cytokines known to induce myofibroblast activation and plays a crucial role in the pathogenesis of HTSs.^41^, ^42^ However, contemporary perspectives suggest that myofibroblasts may lack proficiency in ECM secretion, or potentially never do, as they may not have transitioned through an ECM‐producing state.^1^

To determine whether there is an up‐ or downstream regulatory relationship between the TGF‐β signalling pathway and ALKBH5, we conducted a bidirectional investigation. First, our RNA‐seq analysis revealed that the expression level of TGFB1 in HDFs did not significantly change after ALKBH5 knockdown (Figure S22A). Additionally, we compared TGF‐β levels in the serum and skin tissue homogenates of WT and ALKBH5‐deficient mice using enzyme‐linked immunosorbent assay (ELISA) and found no significant differences between the two groups (Figure S22B). Furthermore, when HDFs were treated with recombinant human TGF‐β1, we detected negligible changes in ALKBH5 expression, suggesting that ALKBH5 is unlikely to be a downstream target of TGF‐β (Figure S22C,D). These findings indicate that the interaction between ALKBH5 and the TGF‐β signalling pathway is likely minimal or non‐existent. Our results indicated that depletion of ALKBH5 directly facilitates the synthesis of ECM components in dermal fibroblasts, independent of both the fibroblast‒myofibroblast transition process and the canonical TGF‐β signalling pathway.

Given the current lack of available agonists for ALKBH5, we explored the use of STM2457, a small‐molecule inhibitor of METTL3,^22^ to counteract the hyper‐methylation in HTS. However, the therapeutic efficacy was limited, likely attributed to the selective regulation of m6A modification on downstream mRNAs by METTL3 and ALKBH5. Therefore, we investigated the therapeutic potential of ALKBH5 through gain‐of‐function assays utilising AAV vectors. Notably, previous studies have also reported AAV‐induced gene therapy for keloid intervention in ex vivo spheroid models.^43^ In view of this, future research and clinical translation efforts may be directed towards the design and development of small molecular agonists specifically targeting ALKBH5, aiming to modulate its activity in a manner that could ameliorate the pathological outcomes associated with fibrotic disorders.

In conclusion, our study revealed the direct regulatory effect of ALKBH5 on ECM components in dermal fibroblasts, which is involved in the pathogenesis of HTSs. Specifically, the absence of ALKBH5 leads to elevated m^6^A modification of *COL3A1*, *COL1A1* and *ELN*, with YTHDF1 recognising their m^6^A modification sites, promoting mRNA stability, and ultimately increasing their expression. We extend the understanding of the epigenetic landscape of HTSs from a novel perspective that reveals the direct regulatory role of m^6^A modification on dermal ECM components. Hopefully, small molecule drugs targeting ALKBH5 or YTHDF1 may be applied in the clinical intervention of cutaneous fibrotic conditions such as HTSs.

## 4 METHODS AND MATERIALS

### 4.1 Clinical samples and ethics statement

Normal skin, normal scar and HTS samples were collected from patients who underwent surgery at the Department of Plastic and Reconstructive Surgery at Shanghai Ninth People's Hospital, Shanghai Jiao Tong University School of Medicine (detailed patient information is summarised in Table S1). Primary HDFs and HSFs were isolated from healthy skin and HTS tissues, respectively. Written informed consent was obtained from all patients, ensuring their understanding of specimen usage in accordance with the Declaration of Helsinki. Approval was granted by the Shanghai Ninth People's Hospital Ethics Committee under permit number SH9H‐2024‐TK265‐1.

### 4.2 Data acquisition and scRNA‐seq data analysis

The dataset used for single‐cell analysis was obtained from the GEO database (GEO: GSE156326 https://www.ncbi.nlm.nih.gov/geo/query/acc.cgi?acc=GSE156326) and includes three normal skin samples and three HTS samples. After the data were processed by the Seurat package (version 4.0.3) in R‐studio (version 4.0.2), they were transformed into Seurat objects, and quality control was applied to each sample's cell‒gene matrix. All sample matrices were analysed to identify 2000 highly variable genes, which were used to integrate all the matrices into one Seurat object, mitigate batch effects, and reduce dimensionality through principal component analysis. Cell clusters were visualised in a UMAP graph, and cell types were annotated according to well‐established marker genes.

RNA‐seq data (GEO: GSE178562; https://www.ncbi.nlm.nih.gov/geo/query/acc.cgi?acc=GSE178562), m^6^A‐seq data (GEO: GSE181540; https://www.ncbi.nlm.nih.gov/geo/query/acc.cgi?acc=GSE181540) and data from the GO database (GO: 0062023 https://amigo.geneontology.org/amigo/search/ontology?q=GO:0062023) were also used for analysis in this study.

### 4.3 Generation of Alkbh5^−/−^ mice

Male *Alkbh5^−/−^* mice aged 6−8 weeks were generated using the CRISPR/Cas9 system as previously described.^15^ In brief, two single‐guide RNAs (sgRNAs) targeting the intronic regions flanking the *Alkbh5* locus were synthesised and transcribed in vitro. These sgRNAs, along with Cas9 protein, were microinjected into zygotes derived from C57BL/6J mice. These manipulated zygotes were then transferred into the oviducts of pseudopregnant Institute of Cancer Research (ICR) females, leading to the generation of F0 mice 19‒21 days post‐transplantation. Genotyping of offspring was performed through PCR amplification and DNA sequencing of tail tissue. To establish a stable F1 generation, F0 mice carrying the desired mutation were bred with wild‐type C57BL/6J mice. All animals were bred and maintained in accordance with institutional guidelines at GemPharmatech Co., Ltd.

### 4.4 Animal scar models and ethics statement

All the procedures for establishing the models were conducted in accordance with the Guide for the Care and Use of Laboratory Animals and were approved by the Committee of Animal Care and Use for Research and Education (CACURE) of Shanghai Jiao Tong University School of Medicine. The ethics permit number for the animal scarring model study was SH9H‐2024‐A469‐SB. Wound scarring was performed as previously described, and scar tissues at the early phase were harvested at POD14, which corresponds to the time point at which immature scars begin to form. The stretch‐induced HTS model was established according to the protocol outlined in the published work by Aarabi et al.,^18^ wherein mechanical stress was consistently applied and discharged upon scar maturation. For the bleomycin‐induced fibrotic scar model, 100 µL of bleomycin solution (B8416, diluted to 1 unit/mL; Sigma‒Aldrich) was administered via intradermal injection at four symmetrically distributed injection sites on the dorsal skin every other day for a duration of 2 weeks.

### 4.5 Histological analysis of the ECM architecture

The specimens were fixed in 4% paraformaldehyde, dehydrated and then paraffin embedded. Sections (5 µm thick) were sliced and stained with Masson's trichrome, Victoria blue or picrosirius red. Quantitative analysis of overall ECM alignment was performed on picrosirius red‐stained images at ×40 magnification using the Orientation J software package.^44^ The local fibre orientation and coherency are presented in coloured map images by visual directional analysis and visualised in the vector field images. Quantification of individual collagen fibre parameters, including length, angle and localised fibre density, was conducted using CT‐FIRE (http://loci.wisc.edu/software/ctfire).^45^ The average fibre metrics for each sample were used for statistical analysis.

### 4.6 RNA m^6^A dot blotting assay

Total RNA was extracted from patients’ skin and scar tissues and quantified using a NanoDrop spectrophotometer. An amount of 1 or 2 µg of RNA was spotted onto a nylon membrane (Biosharp, BS‐NY‐45), crosslinked under ultraviolet light, and blocked, and blocked with 5% milk for 1 h at room temperature. The membranes were then incubated overnight at 4°C with an anti‐m^6^A antibody (1:1000, A19841, ABclonal). The following day, membranes were treated with goat anti‐rabbit immunoglobulin G (IgG)‐HRP secondary antibodies for 1 h at room temperature and visualised using ECL detection (Millipore, WBKLS0100). As a loading control, an equivalent amount of mRNA was spotted on the membranes and stained with .02% methylene blue (Sigma‒Aldrich).

### 4.7 m^6^A RNA methylation assay

Total RNA was extracted from patients’ skin and scar tissues and quantified using a NanoDrop spectrophotometer. The relative change in m^6^A levels compared to total mRNA was assessed using the m^6^A RNA Methylation Assay Kit (Colorimetric) (ab185912), following the manufacturer's protocol. For each sample, 200 ng of RNA was used for analysis.

### 4.8 Collagen gel contraction assay

Cells were seeded in 24‐well plates in 500 µL of collagen suspension. Following collagen gel polymerisation, the gels were released from the plates by gently tilting them. The area of each collagen gel was measured on day 3, and statistical analysis was conducted using Image J software.

### 4.9 MeRIP‐seq and data analysis

MeRIP‐seq was performed as described previously^46^, ^47^ with assistance from Jiayin Biotechnology, Ltd. In brief, total RNA was extracted from HDFs with or without *ALKBH5* knockdown and fragmented into fragments of approximately 100 nucleotides. Approximately 5% of the fragmented RNA was subjected to immunoprecipitation as input, and the remaining RNA was incubated for 2 h at 4°C with an anti‐m^6^A polyclonal antibody (ABE572, Millipore). Following incubation, both the input and immunoprecipitated RNA were used for library construction with the Ovation SoLo RNA‐Seq System Core Kit (NuGEN). Libraries were sequenced on an Illumina NovaSeq 6000 platform with paired‐end reads of 150 bp according to standard protocols. Sequencing was performed with two sets of independent biological replicates. The MeRIP‐seq data are available in the GEO database (GSE264743).

### 4.10 RIP‐qPCR

RNA immunoprecipitation (RIP) was carried out using an RNA immunoprecipitation kit (Geneseed, P0101) following the manufacturer's protocol. Approximately 1.0 × 10^7^ HDFs were treated with 1 mL of RIP lysis buffer. The lysate was then divided into two aliquots: 100 µL reserved as the input sample, and 900 µL was incubated overnight at 4°C with protein A/G magnetic beads conjugated to either a specific antibody or rabbit IgG, in IP buffer containing RNase inhibitors. After incubation, the beads were extensively washed, and the immunoprecipitated RNA was digested, purified and further analysed by qPCR. Details of the primers and antibodies used in the RIP‐qPCR experiments are provided in Tables S4 and S5.

### 4.11 RNA pull‐down

RNA‐protein pull‐down assays were performed using a PureBinding RNA‐Protein pull‐down kit (Geneseed, P0201) following to the manufacturer's protocol. Biotin‐labelled single‐stranded RNA (ssRNA) probes were synthesised in vitro by Sangon Biotin (Shanghai) Co., Ltd. The cell lysate was resuspended and homogenised in a standard lysis buffer as recommended. Ten percent of each sample was reserved as the input control. Subsequently, 100 pmol of RNA probes and 50 µL of magnetic beads were incubated with the lysate at 4°C for 1 h with rotation. The proteins bound to the RNA probes were eluted, and both the eluted protein and input samples were diluted in sodium dodecyl sulfatepolyacrylamide gel electrophoresis (SDS‒PAGE) loading buffer for Western blot analysis. The sequences of the ssRNA probes used are detailed in Table S6.

### 4.12 Detection of RNA half‐life

Approximately 5 × 10^5^ HDFs were seeded per well in six‐well plates. After transfection with the appropriate siRNA, actinomycin‐D (10 µg/mL, HY‐17559, MCE) was added to the cells, and total RNA was extracted at 2, 4, 6 and 8 h for qRT‐PCR to quantify the relative abundance of the remaining mRNAs. The half‐life of the RNA was calculated by Prism GraphPad 9.0 (GraphPad Software, Inc.).

### 4.13 Adeno‐associated virus vector administration

Gain‐of‐function experiments were performed with *Alkbh5*‐encoding AAVs of serotype 9 (AAV9‐*Alkbh5*). The AAV vector and its negative control were obtained from HanBio Co., Ltd. Male C57BL/6J mice aged 6−8 weeks were purchased from Gempharmatech Co., Ltd. and were administered via intradermal injection under anesthesia. Specifically, intradermal injections were performed using AAV vectors (1.5 × 10^12^ vg/mL) or .9% saline (blank control) for 3 days from POD3, when the re‐epithelialisation procedure had been completed. A total volume of 100 µL of AAV vectors or saline was injected at 4−6 symmetrically distributed injection sites on the scarring area for each mouse each day.

### 4.14 TGF‐β1 enzyme‐linked immunosorbent assay

The levels of TGF‐β in serum and skin tissue homogenates were determined by TGF‐β1 ELISA kit (EMC107b.48, NeoBioscience Technology Co., Ltd.), according to the manufacturer's instructions.

### 4.15 Statistics analysis

Statistical analysis was performed by R‐studio (R 4.0.2) and GraphPad Prism 9.0. The data are presented as the means ± standard deviations, and statistical significance is indicated by asterisks as described in the figure legends (^*^ *p *< .05, ^**^ *p *< .01, ^***^ *p *< .001, ^****^ *p *< .0001). For comparative analysis, the control group was normalised to 1% or 100%, and comparisons were made to other treatment groups. Quantitative PCR data were analysed using the ΔΔCt method. The correlation between two datasets was evaluated using simple linear regression analysis. Data distribution was assessed with the Shapiro–Wilk test, Kolmogorov–Smirnov test and D'Agostino and Pearson test for normality. For comparisons between two groups, a two‐tailed unpaired Student's *t*‐test was used if the data were normally distributed and variances were similar (*p* > .05, as determined by the *F*‐test). When variances were unequal (*p* < .05, according to the *F*‐test), the two‐tailed unpaired Student's *t*‐test with Welch's correction was applied. The Mann‒Whitney *U*‐test was used for non‐normally distributed data. For comparisons involving three or more groups with homogeneous variances, ANOVA followed by Dunnett's post‐test or Tukey's post hoc test was employed.

## AUTHOR CONTRIBUTIONS

Ruoqing Xu, En Yang and Xin Huang designed and performed the experiments and drafted the manuscript. Ruoqing Xu, Hsin Liang and Shenying Luo were responsible for samples and patients’ information collection and data analysis. Ruoqing Xu, Yunhan Liu and Yimin Khoong discussed and participated in data interpretation. Xin Huang, Yixuan Zhao and Tao Zan revised and approved the manuscript. Tao Zan (lead surgeon) and Haizhou Li developed the protocol for surgery and patient care; coordinated the research team; and reviewed the manuscript. All the authors reviewed and approved the final version of the manuscript.

## CONFLICT OF INTEREST STATEMENT

The authors declare they have no conflicts of interest.

## Supporting information

Supporting Information

Supporting Information

Supporting Information

## Data Availability

RNA‐seq and MeRIP‐seq data have been deposited in the NCBI GEO database under accession codes GSE264515 (https://www.ncbi.nlm.nih.gov/geo/query/acc.cgi?acc=GSE264515) and GSE264743 (https://www.ncbi.nlm.nih.gov/geo/query/acc.cgi?acc=GSE264743), respectively. All the other data supporting the key findings of this study are available within the article and supplemental information files or from the corresponding author upon reasonable request. Values for all data points in graphs are reported in the Supporting Information Values file.
