# Supplementary material for: ALKBH5‐mediated m6A demethylation ameliorates extracellular matrix deposition in cutaneous pathological fibrosis
Source: Clin Transl Med. 2024 Sep 4;14(9):e70016. doi: 10.1002/ctm2.70016 (PMC11374695; doi:10.1002/ctm2.70016)

**Supplemental material**

**
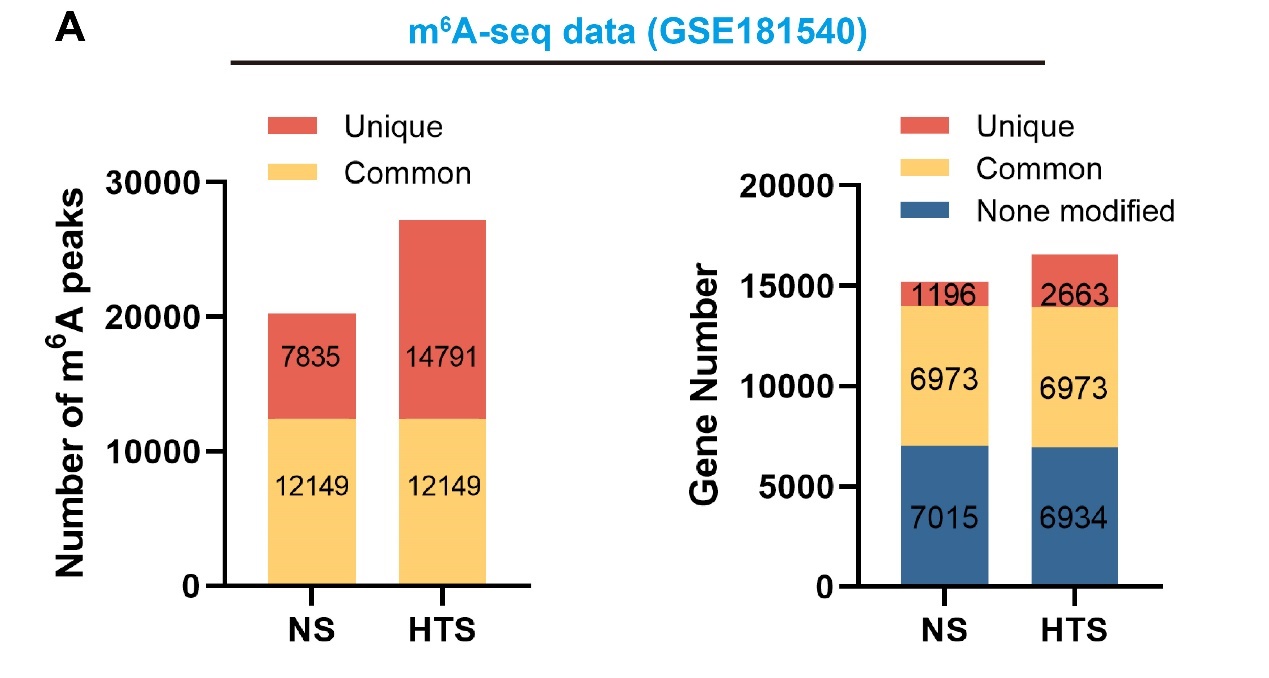
**

**Figure S1. The m^6^A-seq dataset showed elevated m^6^A modification levels in HTS lesions**

(A) The m^6^A-seq dataset shows the numbers of HTS-unique, normal skin-unique, and common m^6^A peaks (left panel) and m^6^A-modified genes.


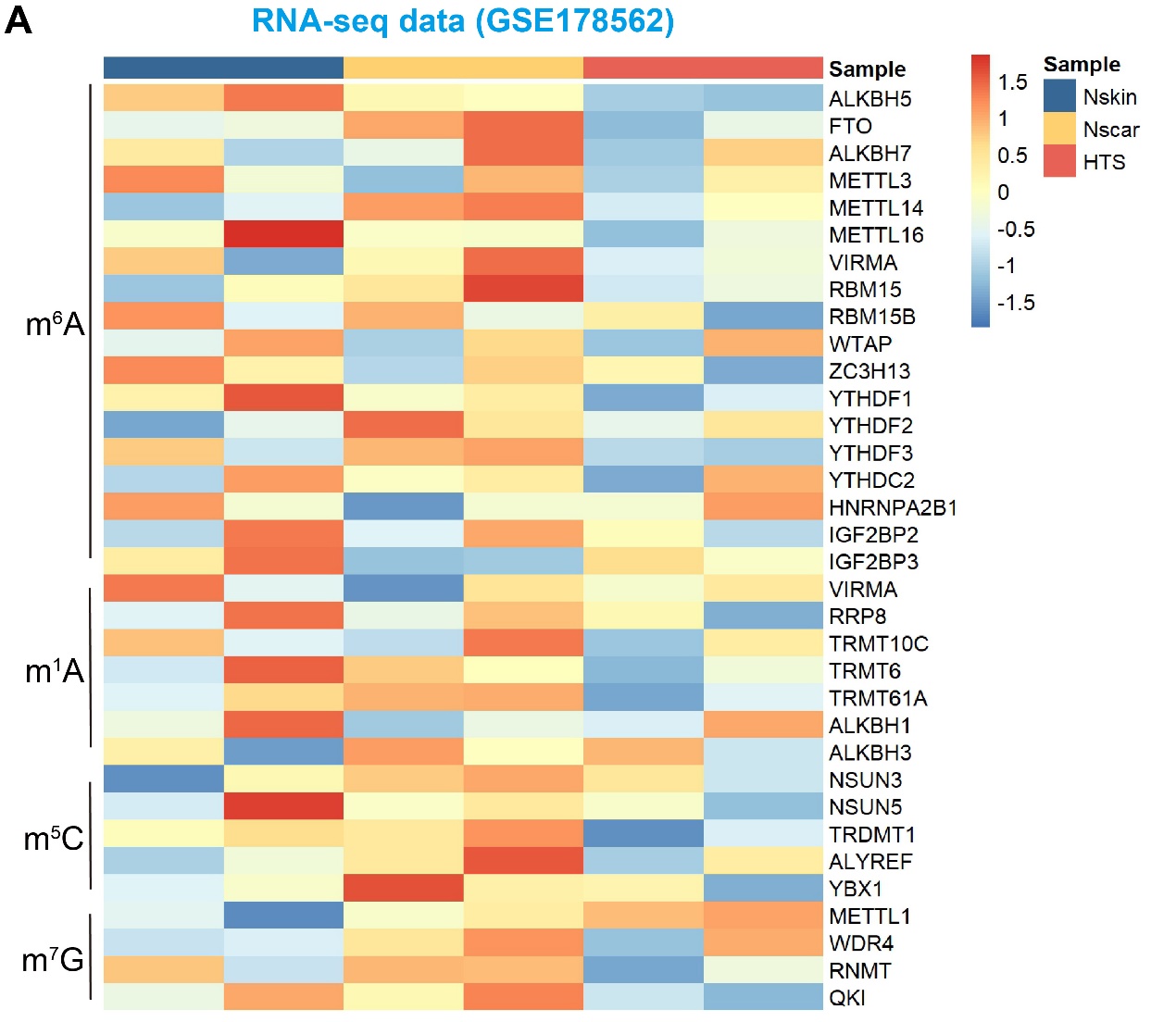


**Figure S2. Expression of RNA modifiers in HTSs**

(A) Heatmap depicting the expression levels of common RNA modifiers involved in m^1^A, m^5^C, and m^7^G modification in normal skin, normal scar tissue and HTS according to RNA-seq data (GEO178562).


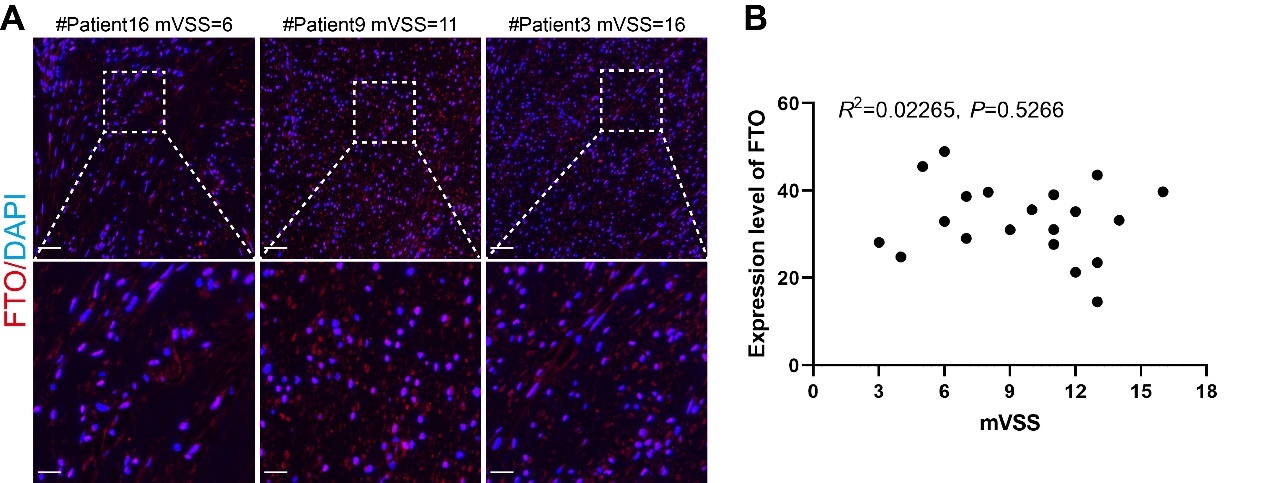


**Figure S3. Correlation analysis of FTO and the mVSS**

(A) FTO expression levels in the dermis of mild, moderate and severe HTSs were visualized by IF. Scale bar: left panel, 100 μm; right panel, 20 μm. (B) Pearson’s *R* correlation plot of the expression level of *FTO* and the mVSS score (n=20). mVSS, modified Vancouver Scar Scale.


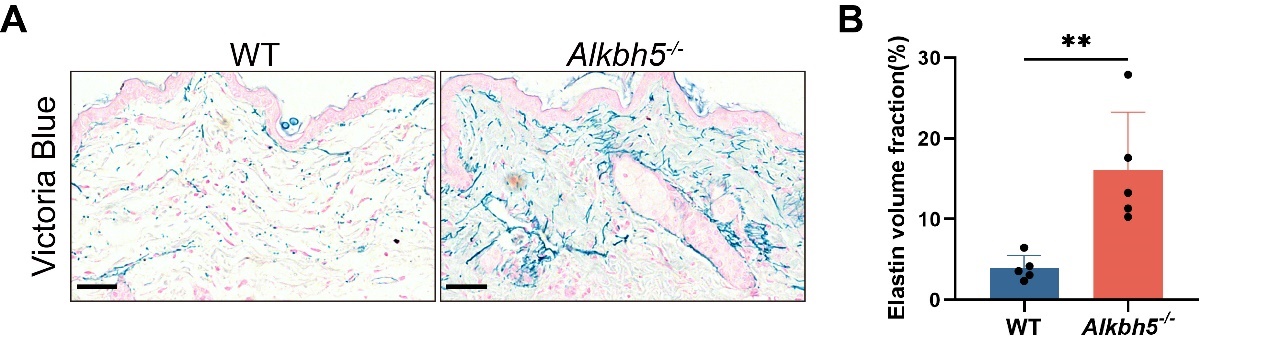


**Figure S4. Expression of ELN in *Alkbh5^-/-^* mice**

(A) Elastic fibres in the dermis were specifically stained with Victoria blue. Scale bar: 50 μm. (B) Quantitative analysis of the elastin volume. The data are presented as the mean ± SD. ***P*<0.01.


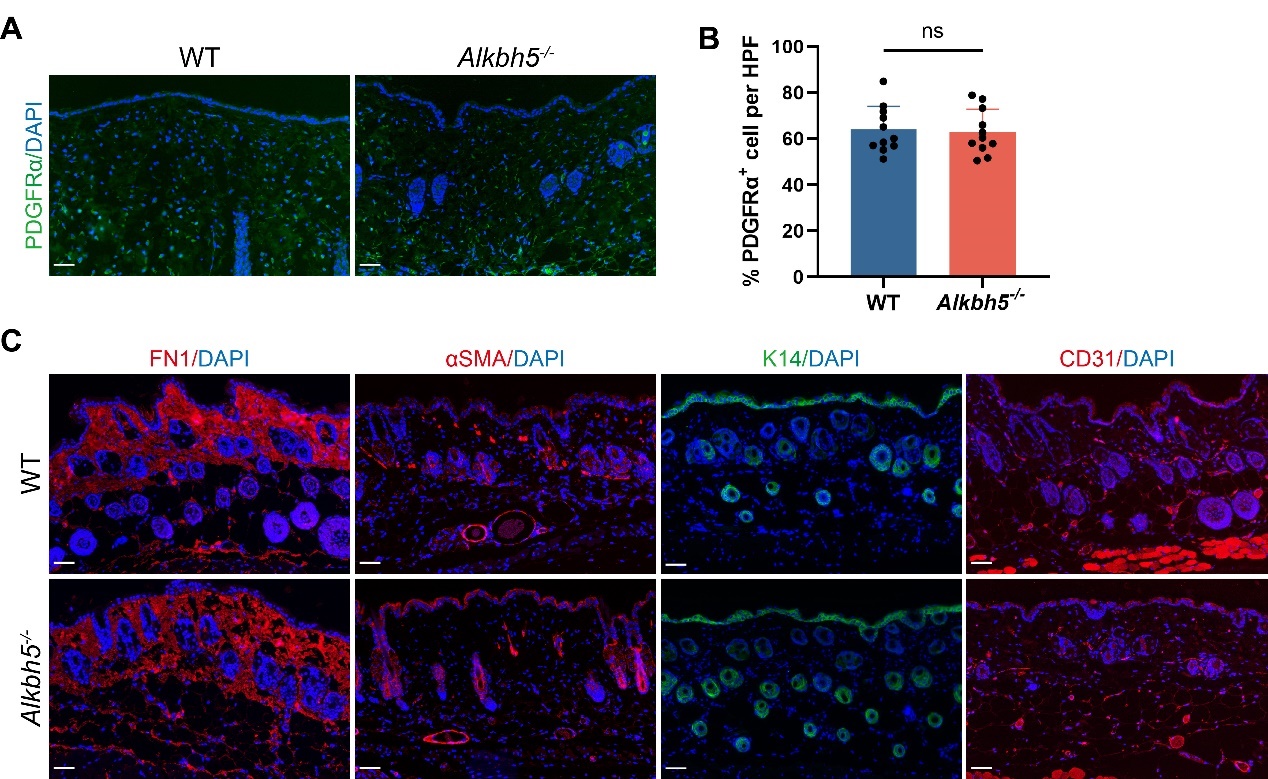


**Figure S5. Expression of other skin components in *Alkbh5^-/-^*mice**

(A) PDGFRα (a marker of murine fibroblasts) expression in the skin of WT and *Alkbh5^-/-^*mice was visualized by immunofluorescence. Scale bar: 200 μm. (B) Statistical analysis of the percentage of PDGFRα^+^ cells (counted as fibroblasts) per HPF. The data are presented as the mean ± SD. ns, not significant. (C) Immunofluorescence images showing the expression of FN1, αSMA, K14 (a marker of keratinocytes), and CD31 (a marker of vascular endothelial cells). Scale bar: 200 μm.


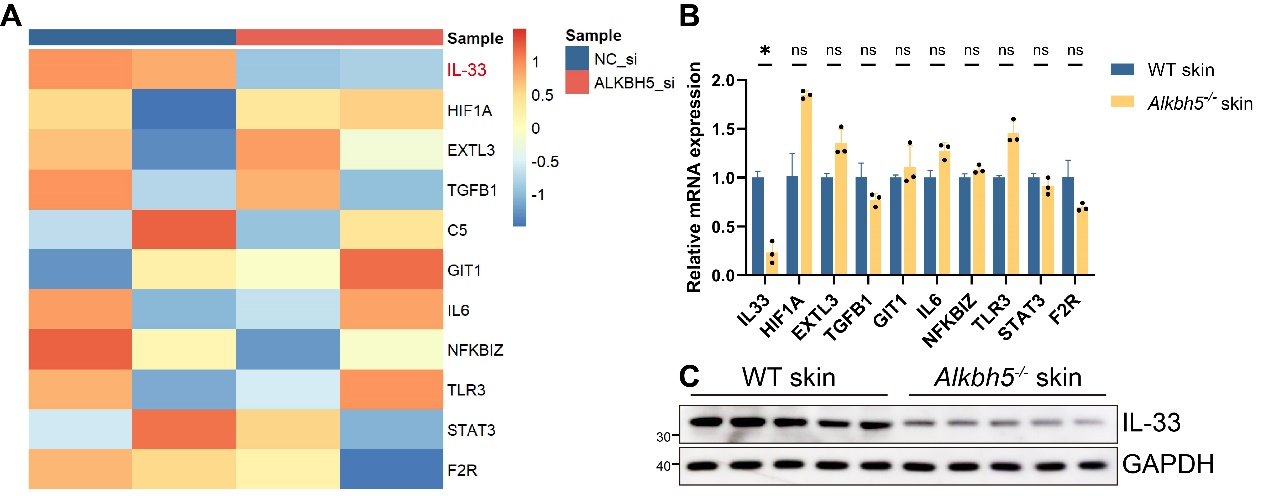


**Figure S6. Immune landscape of ALKBH5-dificient skin**

(A) Heatmap exhibiting the expression levels of genes involved in inflammatory response to wounding. (B) The expression levels of genes in skin tissues of WT and *Alkbh5^-/^*^-^ mice were determined by qRT-PCR. The data are presented as the mean ± SD. ns, not significant; **P*<0.05. (C) Decreased IL-33 expression in skin tissues of *Alkbh5^-/^*^-^ mice was confirmed by WB. The experiments were performed in triplicate.


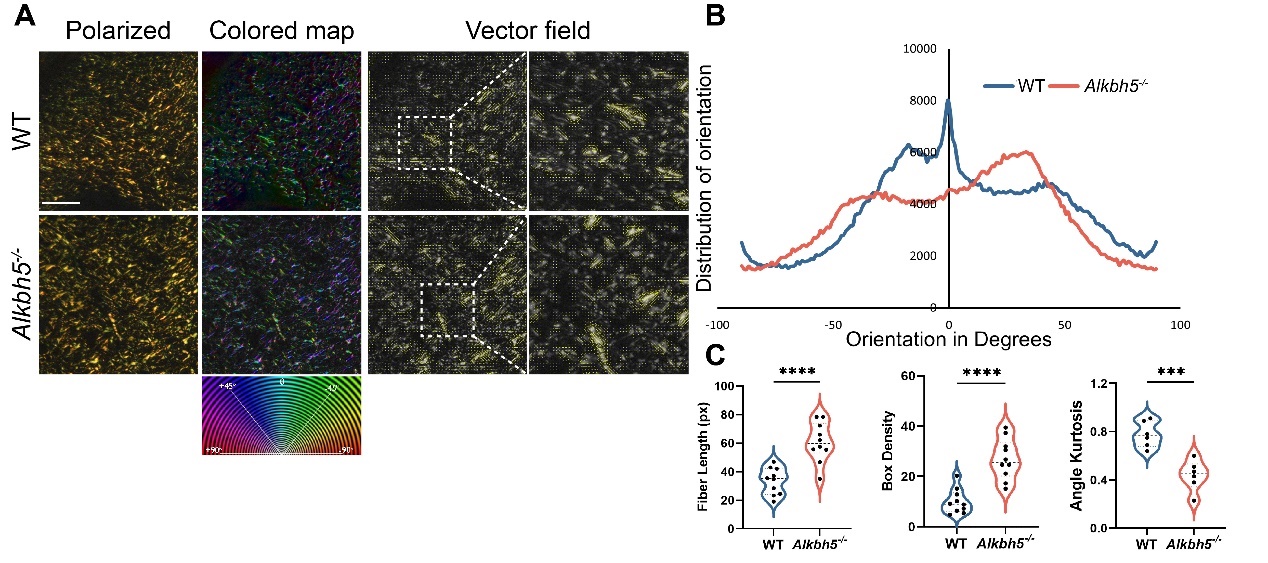


**Figure S7. Orientation analysis of collagen fibres in the wound scarring model**

(A) Fibre orientation analysis was conducted on picrosirius red-stained images under polarized light with Orientation J software. The coloured map and vector field panel visualize the local orientation, coherency and density of the fibres. Scale bar: 100 μm. (B) Quantitative analysis of the distribution of orientations by Orientation J software. (C) Quantification of the different collagen fibre network characteristics, fibre length, box density, and angle kurtosis using the software algorithm CT-FIRE. The data are presented as the mean ± SD. ****P*<0.001, *****P*<0.0001.


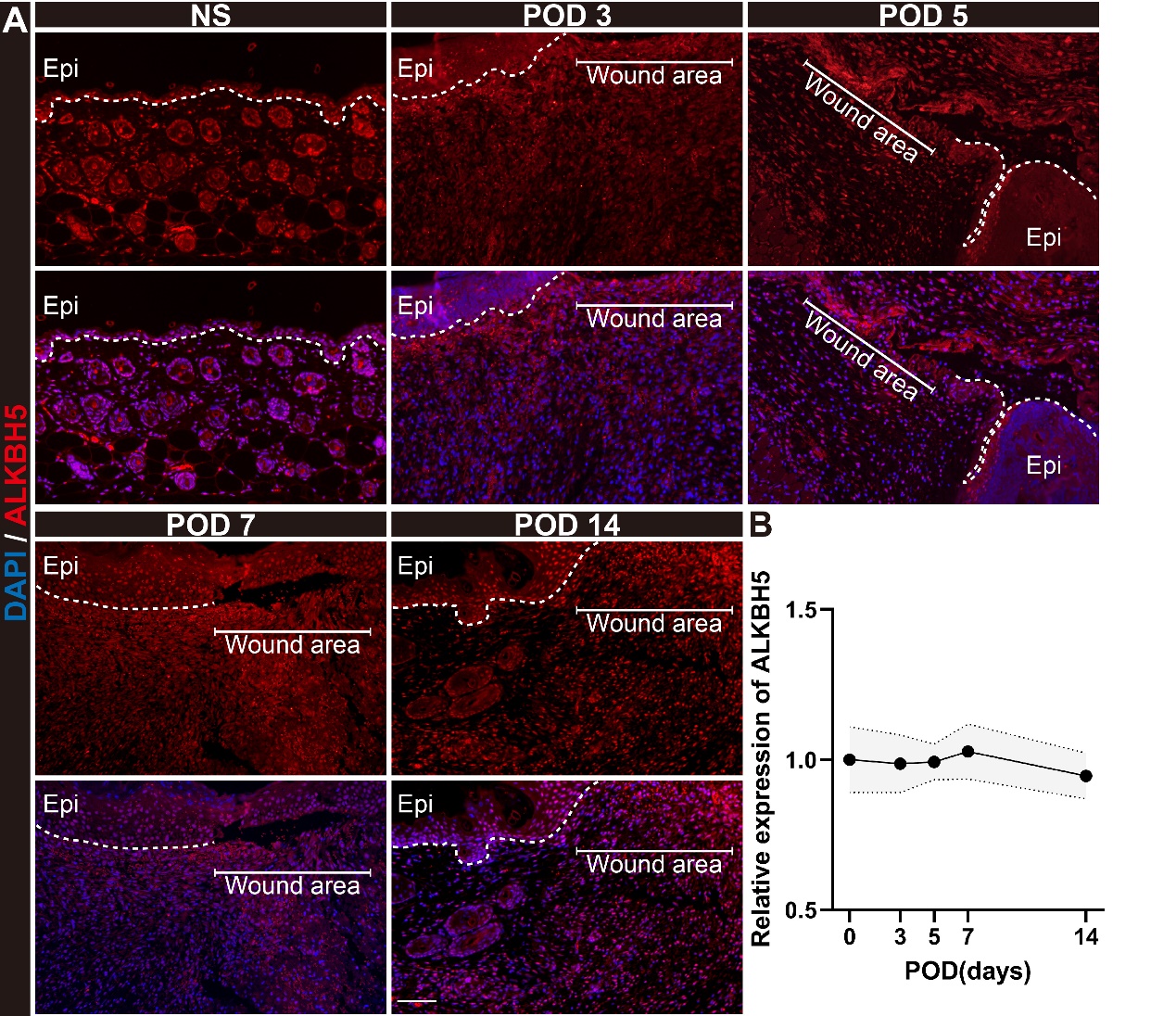


**Figure S8. Dynamic changes in ALKBH5 expression in dermis throughout scar formation**

(A-B) The relative expression levels of ALKBH5 and during physiological scar repair on POD3, POD5, POD7, and POD14 were detected by immunofluorescence staining. Dotted lines denote epidermal boundaries, line segments indicate wound areas. Scale bar: 100 μm. NS, normal skin; Epi, epidermis; POD, post-operation day.


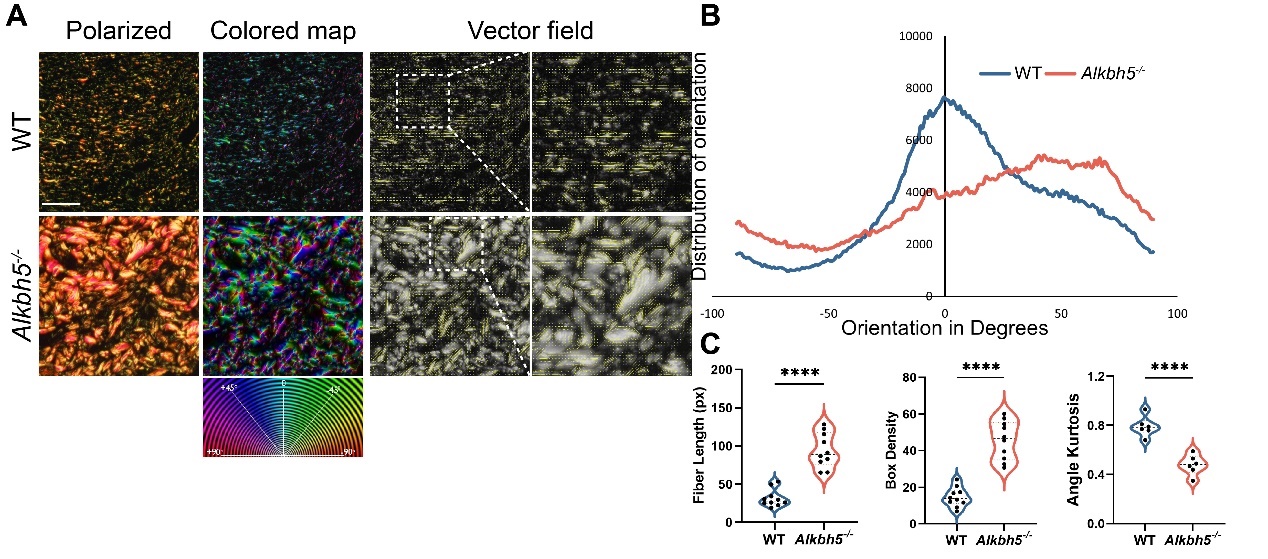


**Figure S9. Orientation analysis of collagen fibres in the mechanical stretch-induced HTS model**

(A) Fibre orientation analysis was conducted on picrosirius red-stained images under polarized light with Orientation J software. The coloured map and vector field panel visualize the local orientation, coherency and density of the fibres. Scale bar: 100 μm. (B) Quantitative analysis of the distribution of orientations by Orientation J software. (C) Quantification of the different collagen fibre network characteristics, fibre length, box density, and angle kurtosis using the software algorithm CT-FIRE. The data are presented as the mean ± SD. *****P*<0.0001.


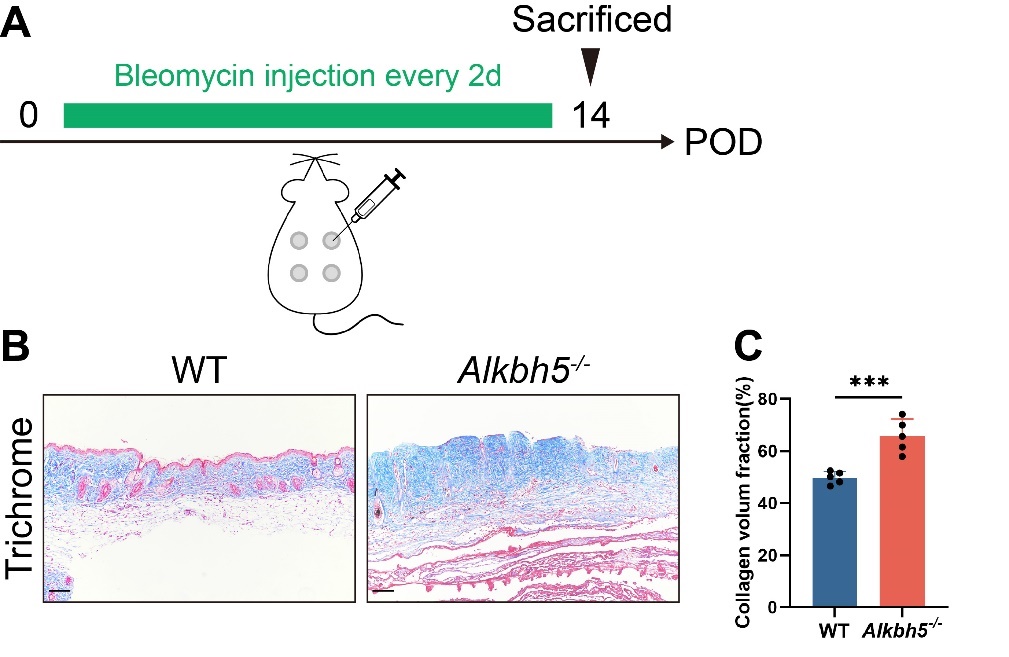


**Figure S10. Bleomycin-induced fibrotic scar model**

(A) Study design of the bleomycin-induced fibrotic scar model (n=5 biologically independent animals). (B) Representative images of Masson’s trichrome-stained sections of the fibrotic scar area. Scale bar: 200 μm. (C) Quantification of the CVF in fibrotic scar tissues. The data are presented as the mean ± SD. ****P*<0.001.


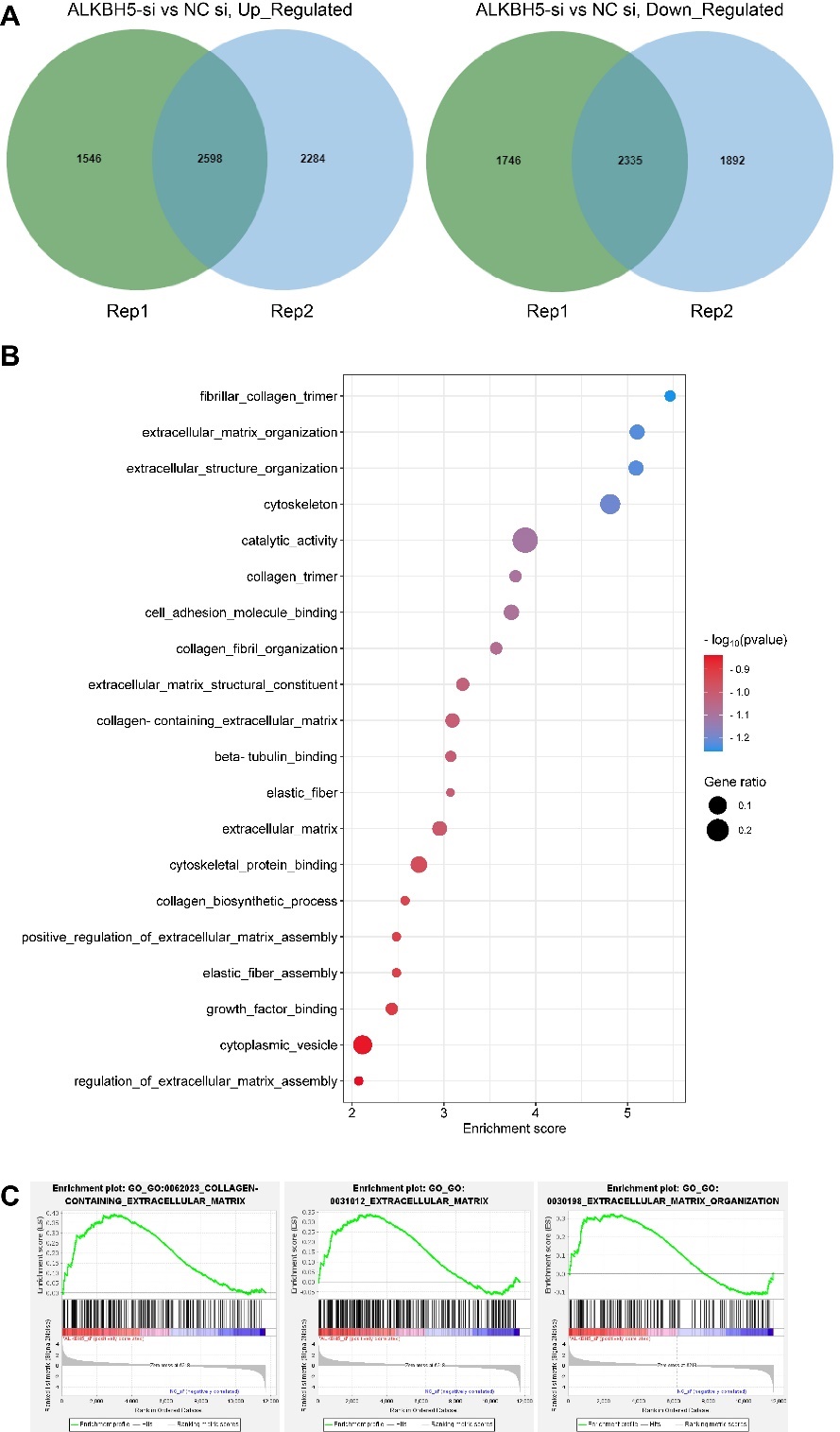


**Fig****ure S11. Gene expression features of ALKBH5 knockdown HDFs**

(A) Venn diagram showing 2598 upregulated and 2335 downregulated genes in ALKBH5 knockdown HDFs identified in biological duplicates via RNA-seq. (B) GO analysis was performed and revealed the functions of the upregulated DEGs. (C) GSEA plots evaluating the changes in the ECM in HDFs with or without ALKBH5 knockdown.


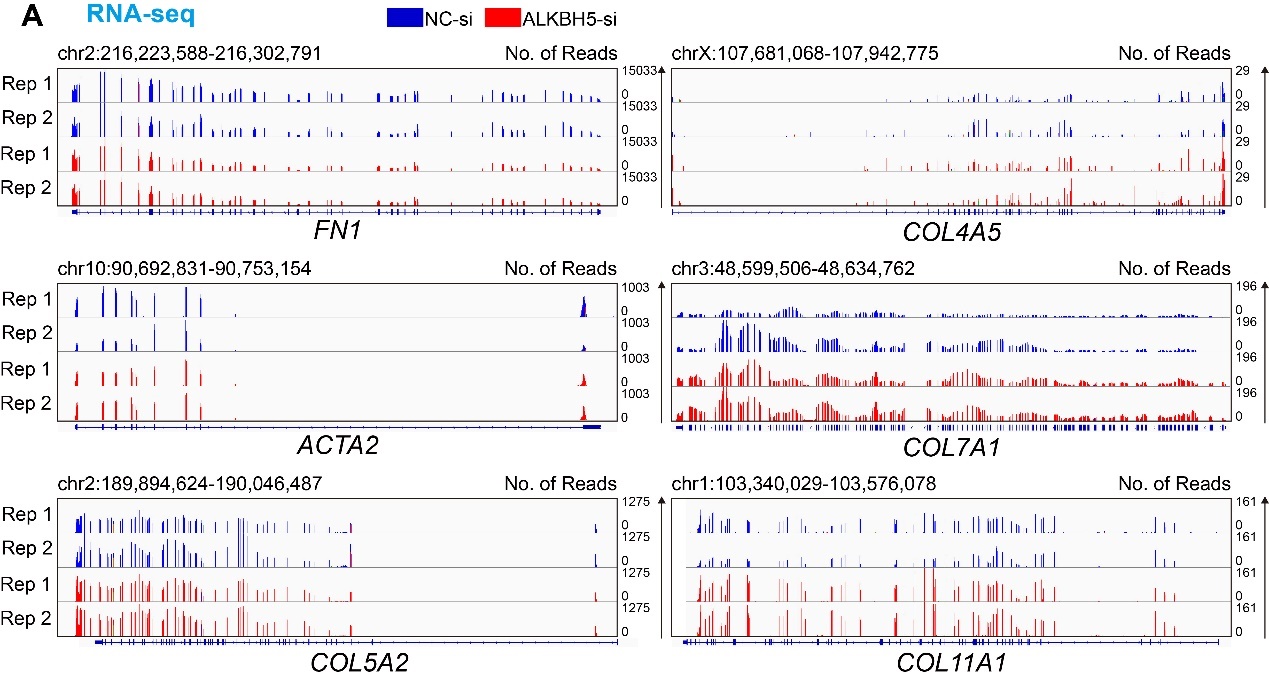


**Figure S12. Expression of other ECM components in ALKBH5 knockdown HDFs**

(A) IGV tracks of dermal ECM components (*FN1*, *ACTA2*, *COL5A2*, *COL4A5*, *COL7A1*, and *COL11A1*) according to RNA-seq analysis of ALKBH5 knockdown or control HDFs. The experiments were performed in duplicate.


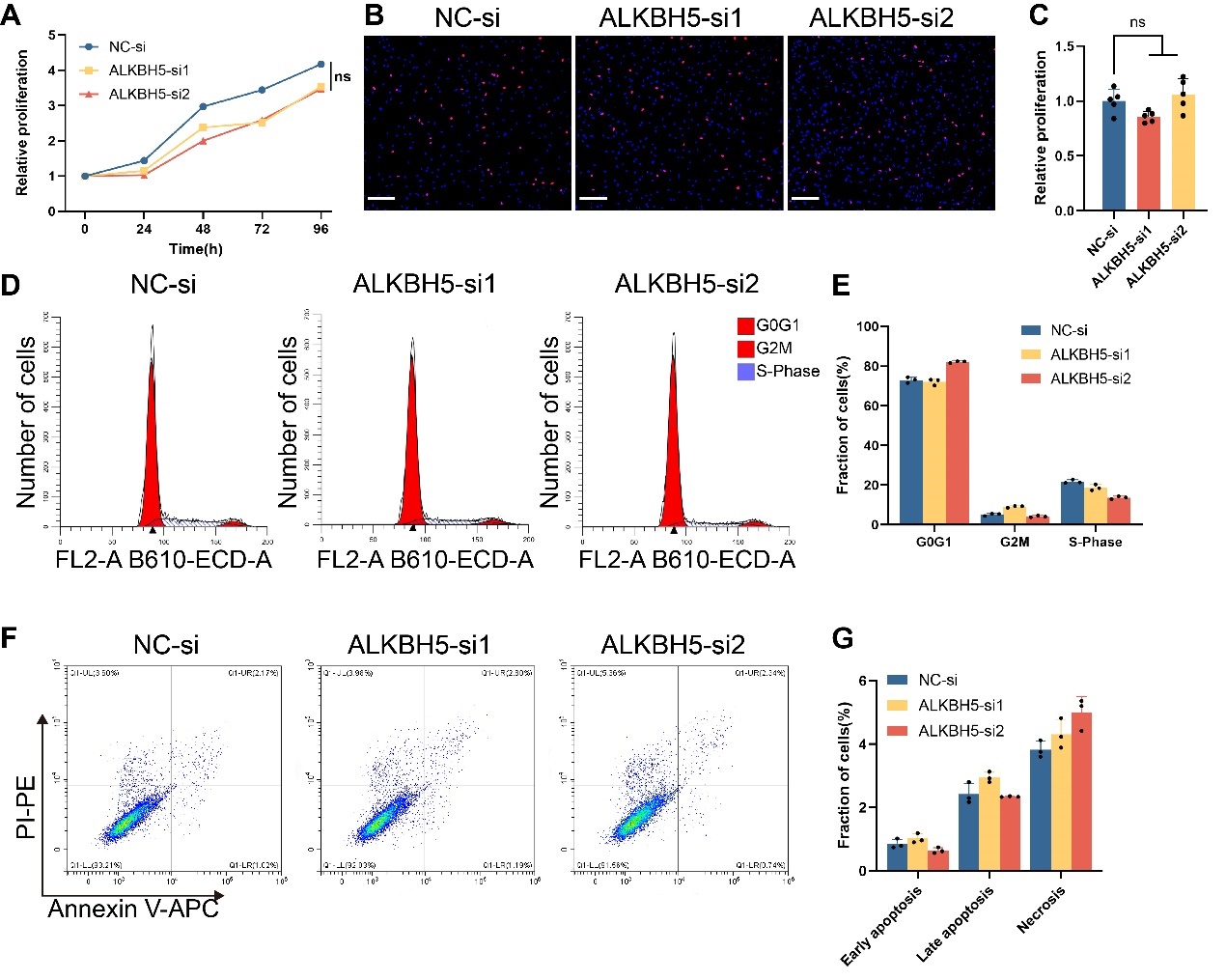


**Figure S13. Inhibition of ALKBH5 showed no effect on HDFs proliferation.**

(A) A CCK-8 assay was performed to assess the proliferation of HDFs with or without ALKBH5 knockdown. The experiments were performed in triplicate. ns, not significant. (B-C) Proliferation of ALKBH5 knockdown or control HDFs was analysed by an EdU staining assay. Scale bar: 100 μm. The data are presented as the mean ± SD. ns, not significant. (D-E) The cell cycle distribution of HDFs with or without ALKBH5 knockdown was analysed by flow cytometry. The experiments were performed in triplicate. The data are presented as the mean ± SD. (F-G) The apoptosis rate of ALKBH5 knockdown or control HDFs was analysed by flow cytometry. The experiments were performed in triplicate. The data are presented as the mean ± SD.


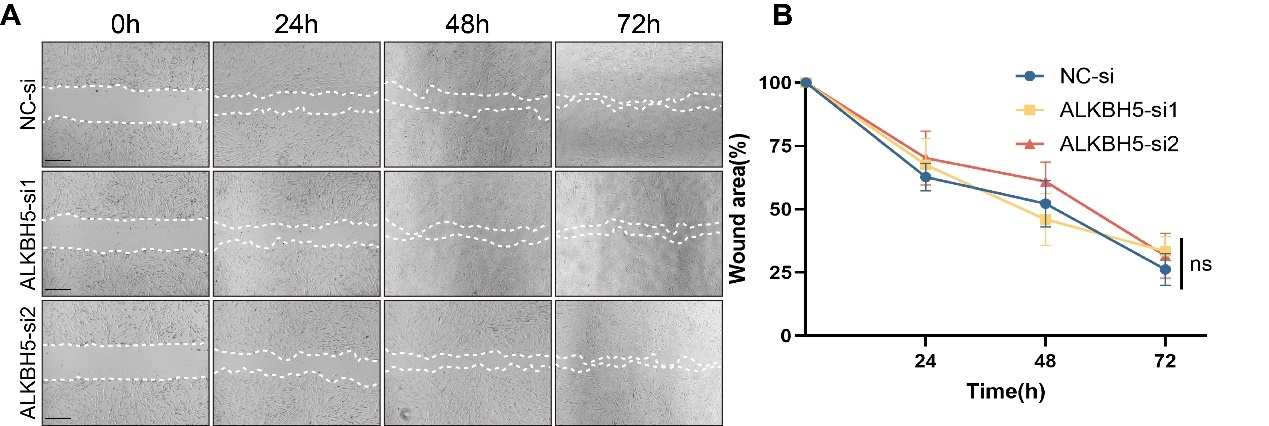


**Figure S14. The migration of ALKBH5 knockdown HDFs**

(A) The long-term migratory ability of ALKBH5 knockdown or control HDFs was evaluated with wound healing assays. The dashed lines outline the edges of the cells. Scale bar: 100 μm. (B) Quantitation of cell migration is presented as the mean ± SD. ns, not significant.


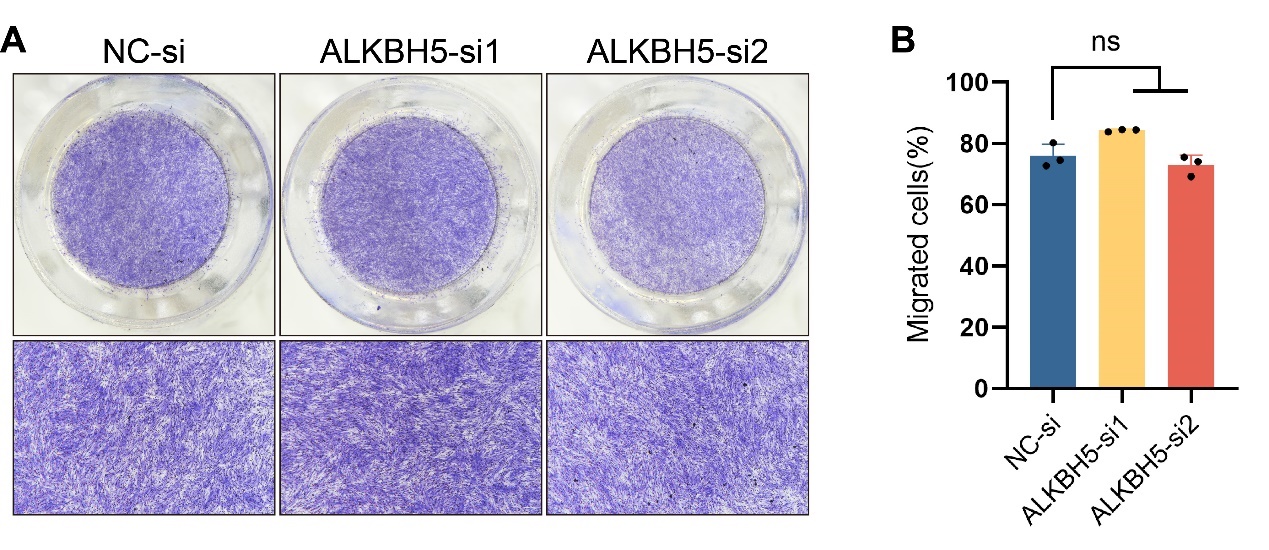


**Figure S15. The invasive function of ALKBH5 knockdown HDFs**

(A) The invasive ability of ALKBH5 knockdown or control HDFs was analysed by Transwell assays (top, global appearance; bottom, local site). (B) Quantitation of cell invasion is presented as the mean ± SD. ns, not significant.


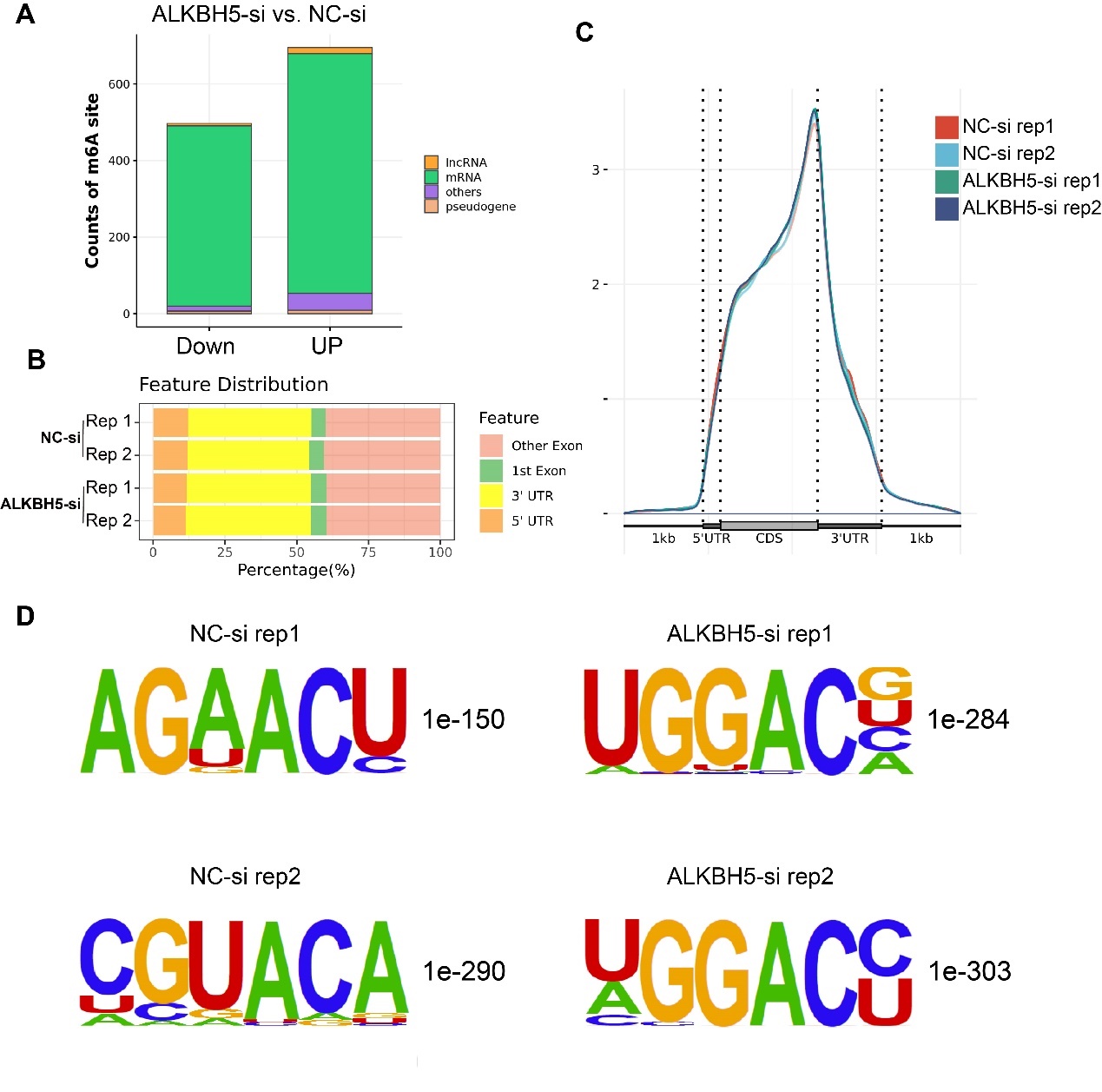


**Figure S16. Genome-wide mapping of m^6^A modifications in HDFs**

(A-B) The stacked bar indicates the distribution of m^6^A modifications in different RNA categories and regions. (C) The distribution of m6A sites along the length of mRNA transcripts. (D) Top enriched motifs within m^6^A peaks that were identified in HDFs with or without ALKBH5 knockdown.


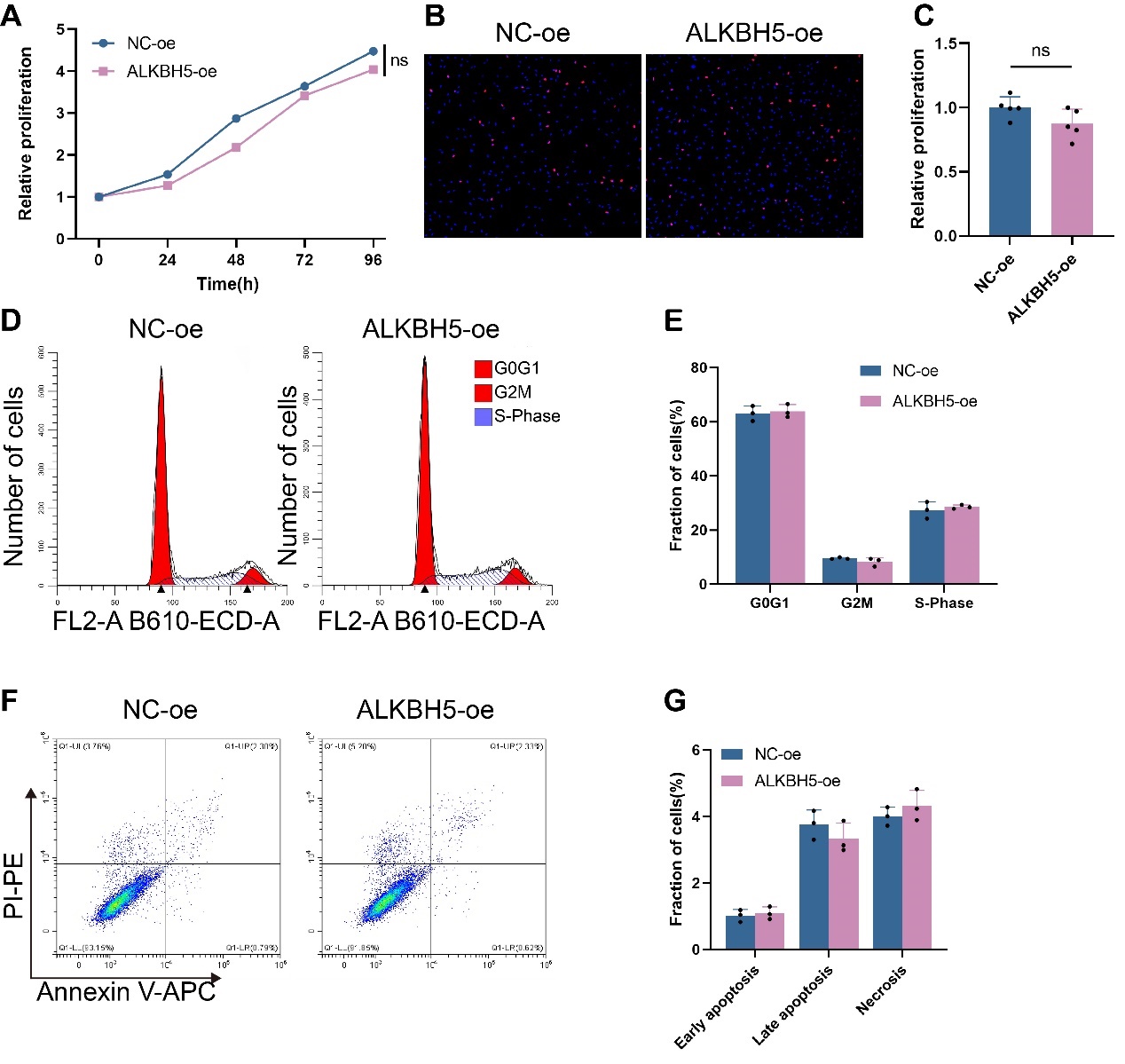


**Figure S17. Exogenous overexpression of ALKBH5 had no effect on HSFs proliferation.**

(A) A CCK-8 assay was performed to assess the proliferation of HSFs with or without ALKBH5 overexpression. The experiments were performed in triplicate. ns, not significant. (B-C) Proliferation of *ALKBH5*-overexpression or control HSFs was analysed by an EdU staining assay. Scale bar: 100 μm. The data are presented as the mean ± SD. ns, not significant. (D-E) The cell cycle distribution of HSFs with or without ALKBH5 overexpression was analysed by flow cytometry. The experiments were performed in triplicate. The data are presented as the mean ± SD. (F-G) The apoptosis rate of ALKBH5-overexpressing or control HDFs was analysed by flow cytometry. The experiments were performed in triplicate. The data are presented as the mean ± SD.


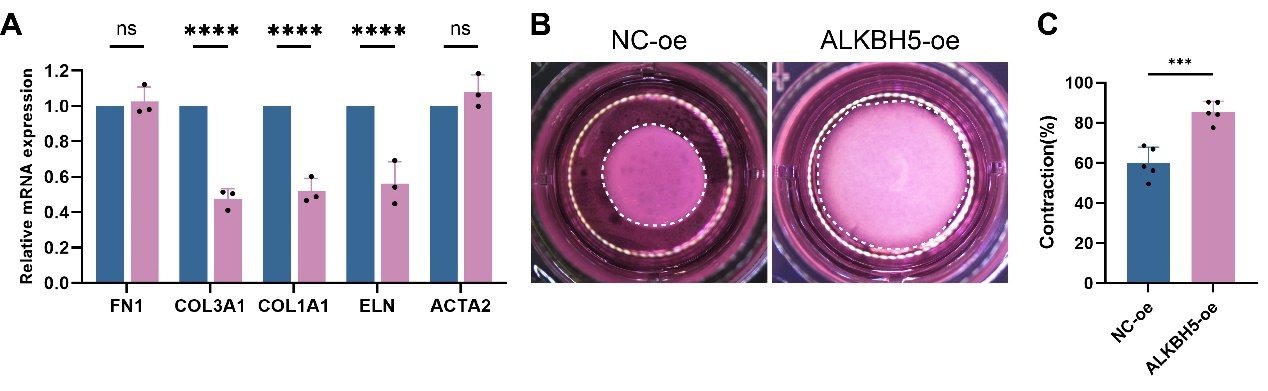


**Figure S18. Changes in the ECM deposition and contraction of ALKBH5-overexpressing HSFs**

(A) The expression levels of major ECM components (*COL3A1*, *COL1A1*, *ELN*, *FN1* and *ACTA2*) in *ALKBH5*-overexpressed and control HSFs were measured by qRT-PCR. The data are presented as the mean ± SD. ns, not significant, *****P*<0.0001. (B-C) Images and quantification of collagen gel contraction assays in *ALKBH5*-overexpressed and control HSFs. The dashed lines indicate the area of the collagen gel. The data are presented as the mean ± SD. ****P*<0.001.


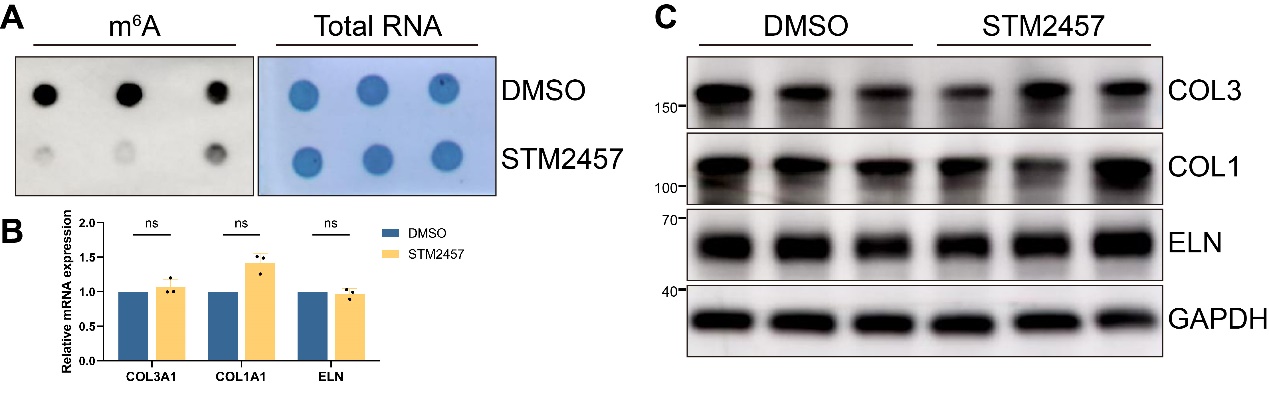


**Figure S19. Therapeutic potential of STM2457 *in vitro***

(A) m6A dot blot showing global m6A modification levels in HSFs treated with or without 5μM STM2457 for 24 hours. Total RNA volume was determined by methylene blue staining, which served as a loading control. The images are representative of experimental triplicates. (B-C) qRT-PCR (B) and WB (C) assays were performed to measure the expression levels of COL3A1, COL1A1 and ELN in HSFs treated with or without STM2457. The experiments were performed in triplicate. ns, not significant.


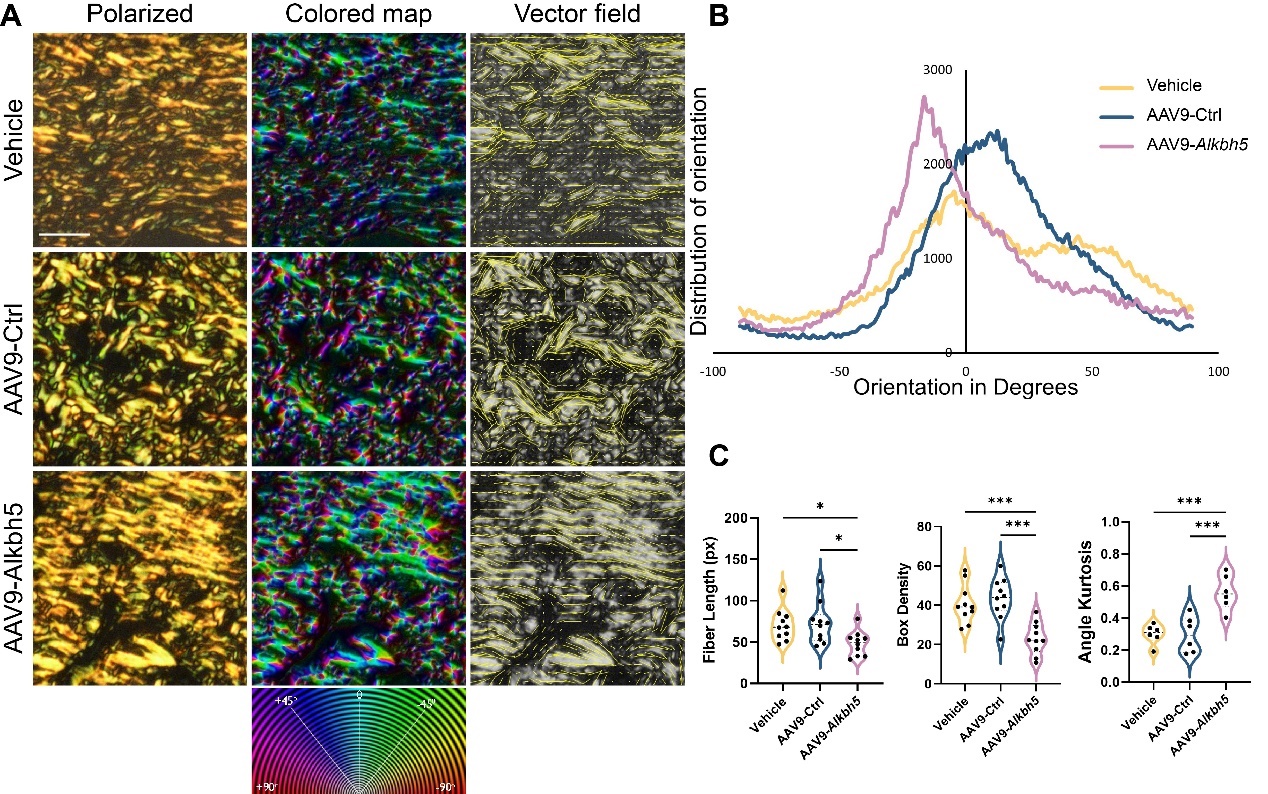


**Figure S20. Orientation analysis of collagen fibres in the AAV vector-treated mechanical stretch-induced HTS model**

(A) Fibre orientation analysis was conducted on picrosirius red-stained images under polarized light with Orientation J software. The coloured map and vector field panel visualize the local orientation, coherency and density of the fibres. Scale bar: 100 μm. (B) Quantitative analysis of the distribution of orientations by Orientation J software. (C) Quantification of the different collagen fibre network characteristics, fibre length, box density, and angle kurtosis using the software algorithm CT-FIRE. The data are presented as the mean ± SD. **P*<0.05, ****P*<0.001.


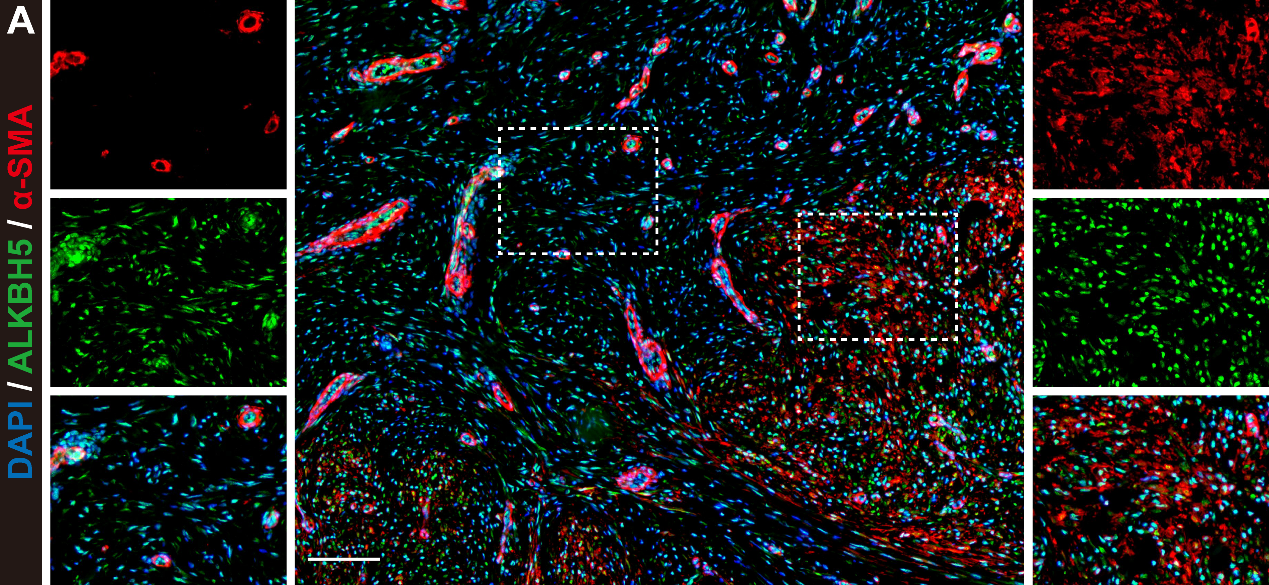


**Figure S21. Co-localization of ALKBH5 and α-SMA in HTS lesions.**

(A) Co-localization of ALKBH5 and α-SMA in HTS lesions were visualized by immunofluorescence. Left panel: α-SMA-negative fibroblasts. Right panel: α-SMA-positive fibroblasts. Scale bar: 200 μm.


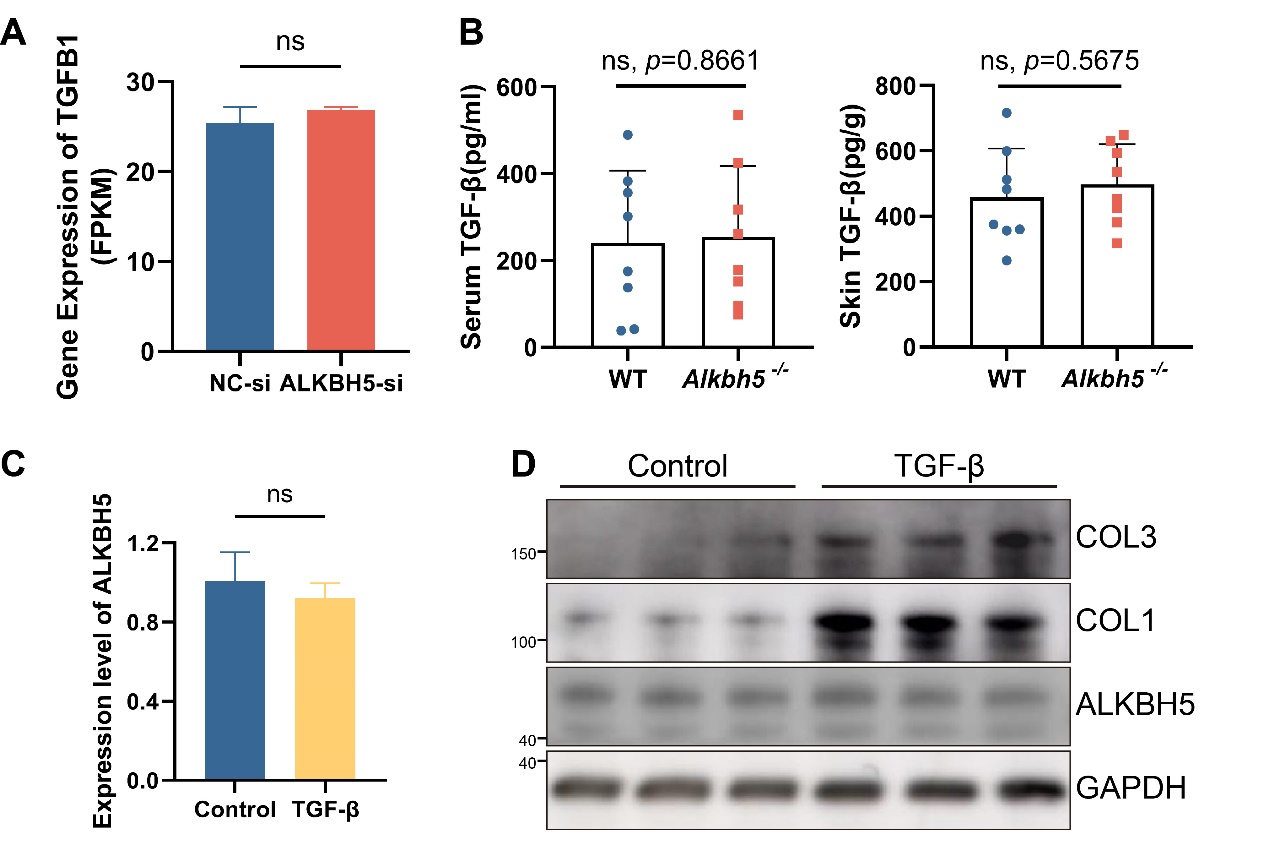


**Figure S22. Mutual regulatory relationship between ALKBH5 and TGF-β.**

(A) RNA-seq data showing the expression level of TGFB1 in the control and ALKBH5 knockdown HDFs. (B) The levels of TGF-β in serum (left panel) and skin tissues homogenates (right panel) of WT and *Alkbh5^-/^*^-^ mice were measured by ELISA kit (n=8). The data are presented as the mean ± SD. ns, not significant. (C-D) qRT-PCR (C) and WB (D) assays were performed to detect the expression level of ALKBH5 in HDFs treated with or without 10ng/mL recombinant human TGF-β1 for 24 hours. The data are presented as the mean ± SD. ns, not significant. The experiments were performed in triplicate.

**Figure S23. Uncropped original western blots**


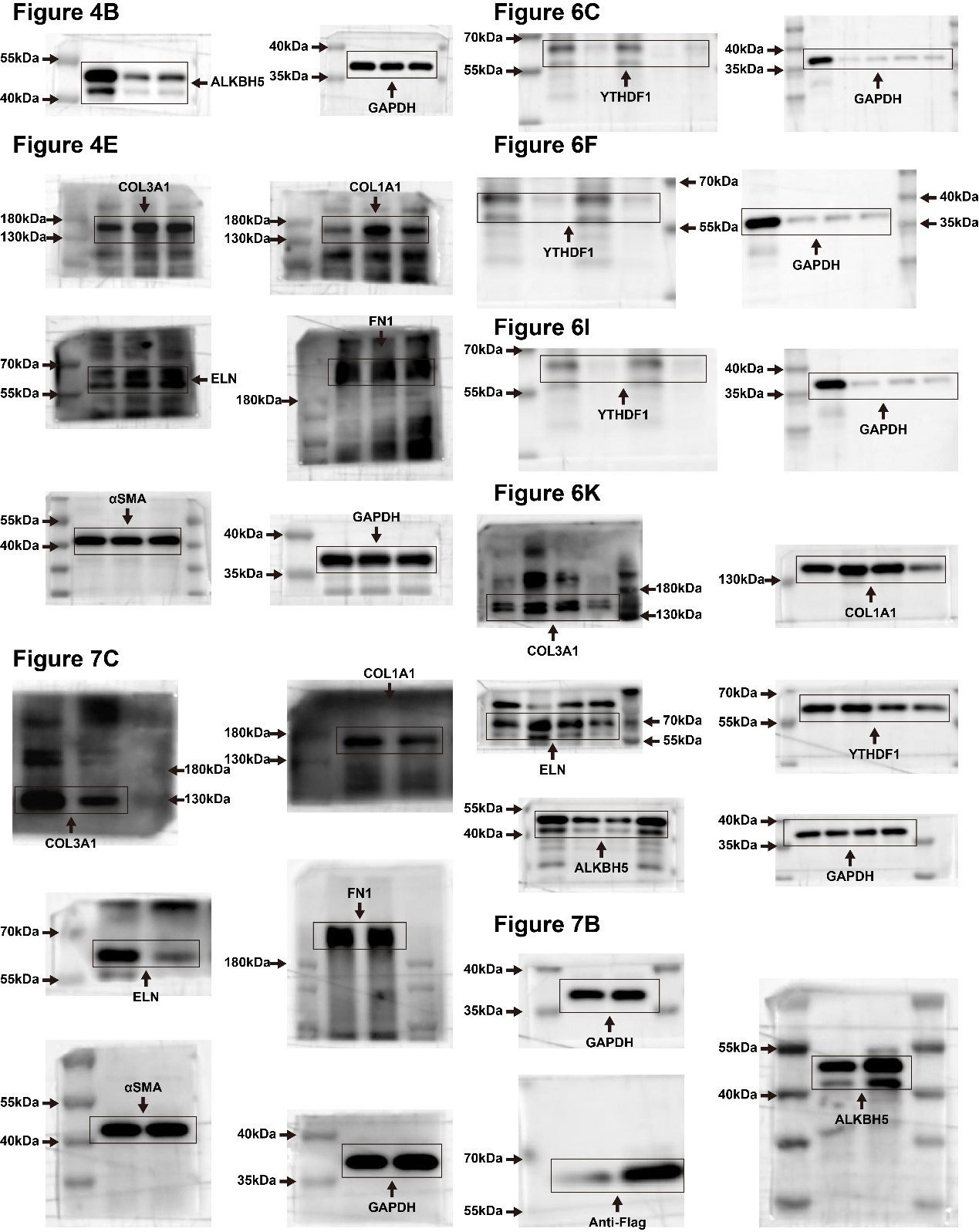

Supplement: Supplementary file 1 — Supporting Information [file CTM2-14-e70016-s001.docx]
