# Supplementary material for: ALKBH5‐mediated m6A demethylation ameliorates extracellular matrix deposition in cutaneous pathological fibrosis
Source: Clin Transl Med. 2024 Sep 4;14(9):e70016. doi: 10.1002/ctm2.70016 (PMC11374695; doi:10.1002/ctm2.70016)
Supplement: Supplementary file 2 — Supporting Information [file CTM2-14-e70016-s003.docx]

**Supporting tables**

**Table S1. Clinical Information of Patients**

| Order | Sex | Age (y) | Causes | Location | Scar Age (m) | mVSS | Experiments and analysis |
| --- | --- | --- | --- | --- | --- | --- | --- |
| HTS1 | F | 22 | Burns | Leg | 60 | 11 | IF (Fig. 1L, 1M, S3B) |
| HTS2 | F | 45 | Surgery | Neck | 240 | 7 | IF (Fig. 1L, 1M, S3B) |
| HTS3 | M | 33 | Burns | Chest | 36 | 16 | Photo presentation (Fig. 1G), Dot blot (Fig. 1H), m^6^A RNA methylation assay (Fig. 1C, 1J), IF (Fig.1L, 1M, S3A, S3B) |
| HTS4 | F | 32 | Trauma | Arm | 36 | 10 | Dot blot (Fig. 1H), m^6^A RNA methylation assay (Fig. 1C, 1J), (Fig. 1L, 1M, S3B) |
| HTS5 | F | 37 | Burns | Face | 8 | 9 | IF (Fig. 1K, 1L, 1M, S3B) |
| HTS6 | M | 19 | Burns | Leg | 24 | 13 | Dot blot (Fig. 1H), m^6^A RNA methylation assay (Fig. 1J), IF (Fig. 1L, 1M, S3B) |
| HTS7 | M | 29 | Trauma | Face | 18 | 12 | Dot blot (Fig. 1H), m^6^A RNA methylation assay (Fig. 1C, 1J), IF (Fig. 1L, 1M, S3B) |
| HTS8 | F | 6 | Burns | Chest | 9 | 4 | Dot blot (Fig. 1H), m^6^A RNA methylation assay (Fig. 1C, 1J), IF (Fig. 1L, 1M, S3B) |
| HTS9 | M | 26 | Burns | Back | 36 | 11 | IF (Fig. 1L, 1M, S3A, S3B) |
| HTS10 | F | 32 | Surgery | Neck | 5 | 12 | IF (Fig. 1L, 1M, S3B) |
| HTS11 | M | 37 | Burns | Face | 10 | 8 | IF (Fig. 1L, 1M, S3B) |
| HTS12 | M | 24 | Trauma | Arm | 48 | 5 | IF (Fig. 1K, 1L, 1M, S3B) |
| HTS13 | M | 42 | Trauma | Face | 30 | 14 | Dot blot (Fig. 1H), m^6^A RNA methylation assay (Fig. 1C, 1J), IF (Fig. 1K, 1L, 1M, S3B) |
| HTS14 | F | 10 | Trauma | Face | 18 | 11 | IF (Fig. 1L, 1M, S3B) |
| HTS15 | M | 52 | Burns | Back | 96 | 7 | IF (Fig. 1L, 1M, S3B) |
| HTS16 | M | 18 | Surgery | Abdomen | 12 | 6 | Dot blot (Fig. 1H), m^6^A RNA methylation assay (Fig. 1J), IF (Fig. 1L, 1M, S3A, S3B) |
| HTS17 | F | 48 | Burns | Face | 18 | 13 | IF (Fig. 1L, 1M, S3B) |
| HTS18 | M | 26 | Burns | Face | 24 | 3 | Photo presentation (Fig. 1G) IF (Fig. 1L, 1M, S3B) |
| HTS19 | F | 44 | Trauma | Arm | 12 | 13 | IF (Fig. 1L, 1M, S3B, S18A) |
| HTS20 | M | 31 | Surgery | Abdomen | 60 | 6 | Dot blot (Fig. 1H), m^6^A RNA methylation assay (Fig. 1J), IF (Fig. 1L, 1M, S3B) |
| HTS21 | F | 25 | Burns | Chest | 12 | 8 | Dot blot (Fig. 1A), cell culture |
| HTS22 | F | 33 | Trauma | Chest | 18 | 10 | Dot blot (Fig. 1A), cell culture |
| HTS23 | M | 44 | Trauma | Back | 24 | 11 | Dot blot (Fig. 1A), cell culture |
| HTS24 | F | 22 | Burns | Neck | 240 | 3 | Dot blot (Fig. 1A, 1H), m^6^A RNA methylation assay (Fig. 1C, 1J) |
| HTS25 | M | 39 | Surgery | Back | 24 | 5 | Dot blot (Fig. 1A, 1H), m^6^A RNA methylation assay (Fig. 1C, 1J) |
| HTS26 | M | 7 | Burns | Neck | 36 | 10 | Dot blot (Fig. 1H), m^6^A RNA methylation assay (Fig. 1C, 1J), cell culture |
| HTS27 | M | 13 | Burns | Face | 120 | 9 | Photo presentation (Fig. 1G), Dot blot (Fig. 1H), cell culture,  m^6^A RNA methylation assay (Fig. 1C, 1J) |
| HTS28 | F | 21 | Surgery | Abdomen | 12 | 11 | Dot blot (Fig. 1H), m^6^A RNA methylation assay (Fig. 1C, 1J), qRT-PCR (Fig. 5C), cell culture |
| HTS29 | M | 11 | Trauma | Face | 18 | 14 | Dot blot (Fig. 1H), m^6^A RNA methylation assay (Fig. 1C, 1J)  qRT-PCR (Fig. 5C), cell culture |
| HTS30 | F | 37 | Surgery | Abdomen | 60 | 15 | Dot blot (Fig. 1H), m^6^A RNA methylation assay (Fig. 1C, 1J)  qRT-PCR (Fig. 5C), cell culture |
| Nscar1 | F | 24 | - | Abdomen | - | - | Dot blot (Fig. 1A), m^6^A RNA methylation assay (Fig. 1C), qRT-PCR (Fig. 5C), cell culture |
| Nscar2 | M | 52 | - | Arm | - | - | Dot blot (Fig. 1A), m^6^A RNA methylation assay (Fig. 1C) |
| Nscar3 | M | 11 | - | Leg | - | - | Dot blot (Fig. 1A), m^6^A RNA methylation assay (Fig. 1C) |
| Nscar4 | F | 32 | - | Abdomen | - | - | qRT-PCR (Fig. 5C) |
| Nscar5 | F | 23 | - | Chest | - | - | qRT-PCR (Fig. 5C) |
| Nscar6 | F | 18 | - | Face | - | - | qRT-PCR (Fig. 5C) |
| Nscar7 | F | 35 | - | Abdomen | - | - | Dot blot (Fig. 1A) |
| Nscar8 | F | 28 | - | Abdomen | - | - | Dot blot (Fig. 1A), m^6^A RNA methylation assay (Fig. 1C) |
| Nscar9 | M | 22 | - | Face | - | - | m^6^A RNA methylation assay (Fig. 1C) |
| Nscar10 | F | 39 | - | Abdomen | - | - | m^6^A RNA methylation assay (Fig. 1C) |
|  |  |  |  |  |  |  |  |
| Nscar11 | F | 18 | - | Chest | - | - | m^6^A RNA methylation assay (Fig. 1C) |
| Nscar12 | M | 20 | - | Arm | - | - | m^6^A RNA methylation assay (Fig. 1C) |
| NS1 | F | 15 | - | Abdomen | - | - | Dot blot (Fig. 1A), m^6^A RNA methylation assay (Fig. 1C) |
| NS2 | F | 27 | - | Chest | - | - | Dot blot (Fig. 1A), m^6^A RNA methylation assay (Fig. 1C) |
| NS3 | M | 33 | - | Chest | - | - | Dot blot (Fig. 1A), qRT-PCR (Fig. 5C) |
| NS4 | M | 4 | - | Foreskin | - | - | qRT-PCR (Fig. 5C), cell culture |
| NS5 | M | 3 | - | Foreskin | - | - | qRT-PCR (Fig. 5C), cell culture |
| NS6 | M | 1 | - | Foreskin | - | - | cell culture |
| NS7 | M | 3 | - | Foreskin | - | - | cell culture |
| NS8 | M | 2 | - | Foreskin | - | - | cell culture |
| NS9 | M | 6 | - | Foreskin | - | - | cell culture |
| NS10 | M | 2 | - | Foreskin | - | - | cell culture |
| NS11 | M | 3 | - | Foreskin | - | - | cell culture |
| NS12 | M | 5 | - | Foreskin | - | - | cell culture |
| NS13 | F | 23 | - | Abdomen | - | - | Dot blot (Fig. 1A), m^6^A RNA methylation assay (Fig. 1C) |
| NS14 | F | 48 | - | Abdomen | - | - | Dot blot (Fig. 1A), m^6^A RNA methylation assay (Fig. 1C) |
| NS15 | M | 13- | - | Abdomen | - | - | m^6^A RNA methylation assay (Fig. 1C) |
| NS16 | M | 42 | - | Chest | - | - | m^6^A RNA methylation assay (Fig. 1C) |
| NS17 | M | 57 | - | Chest | - | - | m^6^A RNA methylation assay (Fig. 1C) |
| NS18 | M | 11 | - | Abdomen | - | - | m^6^A RNA methylation assay (Fig. 1C) |
| NS19 | F | 27 | - | Abdomen | - | - | m^6^A RNA methylation assay (Fig. 1C) |
| NS20 | F | 15 | - | Chest | - | - | m^6^A RNA methylation assay (Fig. 1C) |

Abbreviations: HTS, hypertrophic scar; Nscar, normal scar; NS, normal skin; y, year; m, month; IF, immunofluorescence.

**Table S2. modified Vancouver Scar Scale**

| Items | Feature | Score |
| --- | --- | --- |
| Vascularity | Normal | 0 |
|  | Pink | 1 |
|  | Red | 2 |
|  | Purple | 3 |
| Pigmentation | Normal | 0 |
|  | Hypopigmentation | 1 |
|  | Mixed-pigmentation | 2 |
|  | Hyperpigmentation | 3 |
| Pliability (elasticity) | Normal | 0 |
|  | Supple (flexible with minimal resistance) | 1 |
|  | Yielding (giving way to pressure) | 2 |
|  | Firm (inflexible, not easily moved, resistant to manual pressure) | 3 |
|  | Banding (rope-like tissue that blanches with extension of the scar) | 4 |
|  | Contracture (permanent shortening of scar, producing deformity or distortion) | 5 |
| Height | Flat | 0 |
|  | ＜2 mm | 1 |
|  | 2–5 mm | 2 |
|  | ＞5 mm | 3 |
| Pain | None | 0 |
|  | Occasional | 1 |
|  | Requires medication | 2 |
| Itchiness | None | 0 |
|  | Occasional | 1 |
|  | Requires medication | 2 |

**Table S3. Oligonucleotides used for siRNA expression vector**

| Oligonucleotides name | Sequence (5’‒3’) |
| --- | --- |
| ALKBH5‒si1‒sense | GCUUCAGCUCUGAGAACUATT |
| ALKBH5‒si1‒antisense | UAGUUCUCAGAGCUGAAGCTA |
| ALKBH5‒si2‒sense | GCUGCAAGUUCCAGUUCAATT |
| ALKBH5‒si2‒antisense | UUGAACUGGAACUUGCAGCCG |
| YTHDF1‒si‒sense | GUUCGUUACAUCAGAAGGAUATT |
| YTHDF1‒si‒antisense | UAUCCUUCUGAUGUAACGAACTT |

**Table S4. Primers used in experiments**

| Primer sequences for qRT‒PCR | | | |
| --- | --- | --- | --- |
| GAPDH | Human | Forward | 5’‒ ATGTTGCAACCGGGAAGGA‒3’ |
|  |  | Reverse | 5’‒ CAGGAGCGCAGGGTTAGTC‒3’ |
| ALKBH5 | Human | Forward | 5’‒ATCCTCAGGAAGACAAGATTAG‒3’ |
|  |  | Reverse | 5’‒TTCTCTTCCTTGTCCATCTC‒3’ |
| COL3A1 | Human | Forward | 5’‒AGCCTGGTAAGAATGGTGCC‒3’ |
|  |  | Reverse | 5’‒TCCTTGCCATCTTCGCCTTT‒3’ |
| COL1A1 | Human | Forward | 5’‒GCTTGGTCCACTTGCTTGAA‒3’ |
|  |  | Reverse | 5’‒TTTGGGAAGGAGTGGAGGG‒3’ |
| ELN | Human | Forward | 5’‒TCCTCCTGCTCCTGCTGTCC‒3’ |
|  |  | Reverse | 5’‒TCCAAGGGCTCCGAGACCAG‒3’ |
| FN1 | Human | Forward | 5’‒ TCAGCTTCCTGGCACTTCTG‒3’ |
|  |  | Reverse | 5’‒ TCTTGTCCTACATTCGGCGG‒3’ |
| ACTA2 | Human | Forward | 5’‒ AAAAGACAGCTACGTGGGTGA‒3’ |
|  |  | Reverse | 5’‒ GCCATGTTCTATCGGGTACTTC‒3’ |
| DAG1 | Human | Forward | 5’‒ AGACGGTTCTTCCCCCAGTA‒3’ |
|  |  | Reverse | 5’‒ CCGCCTGAACTGTAGGAGTC‒3’ |
| WNT5B | Human | Forward | 5’‒ GCGAGAAGACTGGAATCAGGG‒3’ |
|  |  | Reverse | 5’‒ TGCACCGGGTTCAAAGCTAA‒3’ |
| COL5A1 | Human | Forward | 5’‒ CTTGGCCCAAAGAAAACCCG‒3’ |
|  |  | Reverse | 5’‒ GTAGGTGACGTTCTGGTGGG‒3’ |
| EMILIN1 | Human | Forward | 5’‒ AGGATCCGGTCATCAGGGAA‒3’ |
|  |  | Reverse | 5’‒ GGAGCTGCGTCTCAGAAGTT‒3’ |

| GAPDH | Mouse | Forward | 5’‒CAGTGGCAAAGTGGAGATTGTTG‒3’ |
| --- | --- | --- | --- |
|  |  | Reverse | 5’‒TCGCTCCTGGAAGATGGTGAT‒3’ |
| IL33 | Mouse | Forward | 5’‒GGGCTCACTGCAGGAAAGT‒3’ |
|  |  | Reverse | 5’‒CGAGACGTCACCCCTTTGAA‒3’ |
| HIF1A | Mouse | Forward | 5’‒CTTGACAAGCTAGCCGGAGG‒3’ |
|  |  | Reverse | 5’‒AAACCATGTCGCCGTCATCT‒3’ |
| EXTL3 | Mouse | Forward | 5’‒TTGTGGGAAGCGGACTTTCA‒3’ |
|  |  | Reverse | 5’‒TTGCCCACTCTGATCACGTC‒3’ |
| TGFB1 | Mouse | Forward | 5’‒CATCCATGACATGAACCGGC ‒3’ |
|  |  | Reverse | 5’‒GAAGTTGGCATGGTAGCCCT ‒3’ |
| GIT1 | Mouse | Forward | 5’‒CTCCTACGCTGCTCCAGATG‒3’ |
|  |  | Reverse | 5’‒TTTGCCGAGATCTGTCAGCC‒3’ |
| IL6 | Mouse | Forward | 5’‒GCCTTCTTGGGACTGATGCT‒3’ |
|  |  | Reverse | 5’‒TGTGACTCCAGCTTATCTCTTGG‒3’ |
| NFKBIZ | Mouse | Forward | 5’‒CGTACTAGTCGCTCTGCCG‒3’ |
|  |  | Reverse | 5’‒GATTCGCTGTGGCGAACATC‒3’ |
| TLR3 | Mouse | Forward | 5’‒TGCGCATATCACAGGCTGAA‒3’ |
|  |  | Reverse | 5’‒AGCCCAGATTATGGGTGCAA‒3’ |
| STAT3 | Mouse | Forward | 5’‒GTGTGACACCATTCATTGATGC‒3’ |
|  |  | Reverse | 5’‒ATCTGCAACCATTCCAGGGT‒3’ |
| F2R | Mouse | Forward | 5’‒TGGAGGGTAGGGCAGTCTAC‒3’ |
|  |  | Reverse | 5’‒CTGGATCGGATACACCACCG‒3’ |
|  |  |  |  |
|  |  |  |  |
|  |  |  |  |

| Primer sequences for RNA Binding Protein Immunoprecipitation (RIP) | | | |  |
| --- | --- | --- | --- | --- |
| COL3A1 | | Human | Forward | 5’‒CGTTGGCCCTGTTTGCTTTT‒3’ |
|  | |  | Reverse | 5’‒AGAACAAGAGGAACACATATGGAGT‒3’ |
| COL1A1 | | Human | Forward | 5’‒AGGTTGGGATGGAGGGAGTTTAC‒3’ |
|  | |  | Reverse | 5’‒GGACGTTGGTGCCCCAGAC‒3’ |
| ELN | | Human | Forward | 5’‒CGTCTCCTCCCCACCGATC‒3’ |
|  | |  | Reverse | 5’‒CAAGGGCAAGGTGGCTATTCC‒3’ |

**Table S5. Antibodies used in experiments**

| Antibodies | Source | Identifier | Dilution |
| --- | --- | --- | --- |
| anti-ALKBH5 | Abcam | ab195377 | IHC-P: 1:200; WB: 1:1000 |
| anti-FTO | Proteintech | Cat No.27226-1-AP | IHC-P: 1:200; WB: 1:1000 |
| anti-COL3A1 | Proteintech | Cat No.22734-1-AP | IHC-P: 1:200; WB: 1:1000 |
| anti-COL1A1 | Proteintech | Cat No.14695-1-AP | IHC-P: 1:200; WB: 1:1000 |
| anti-ELN | Proteintech | Cat No.15257-1-AP | IHC-P: 1:100; WB: 1:1000 |
| anti-FN1 | Proteintech | Cat No.15613-1-AP | IHC-P: 1:200; WB: 1:1000 |
| anti-αSMA | Abcam | ab33625 | IHC-P: 1:400; WB: 1:1000 |
| anti-PDGFRα | Abcam | ab96569 | IHC-P: 1:200 |
| anti-Keratin14 | CST | #48020S | IHC-P: 1:500 |
| anti-CD31 | Abcam | ab182981 | IHC-P: 1:200 |
| anti-Flag | PTMBIO | Cat #PTM-6075 | WB: 1:1000 |
| anti-m^6^A | Abclonal | A19841 | RIP: 5μg; Dot Blot: 1:1000 |
| anti-GAPDH | Proteintech | Cat No.10494-1-AP | WB: 1:2000 |
| anti-YTHDF1 | Proteintech | Cat No.17479-1-AP | RIP: 5μg; WB: 1:1000 |
| anti-YTHDF2 | Proteintech | Cat No.24744-1-AP | RIP: 5μg; WB: 1:1000 |
| anti-YTHDF3 | Proteintech | Cat No.25537-1-AP | RIP: 5μg; WB: 1:1000 |
| Normal rabbit IgG antibody | Millipore | Cat#PP64 | RIP: 5μg; Dot Blot: 1:10000 |
| Anti-rabbit IgG, HRP-linked Antibody | CST | #7074S | WB: 1:2500 |
| Anti-mouse IgG (H+L), F(ab')2 Fragment (Alexa Fluor® 488 Conjugate) | CST | #4408S | IHC-P: 1:400 |
| Anti-rabbit IgG (H+L), F(ab')2 Fragment (Alexa Fluor® 594 Conjugate) | CST | #8889S | IHC-P: 1:400 |
| Anti-mouse IgG (H+L), F(ab')2 Fragment (Alexa Fluor® 594 Conjugate) | CST | #8890 | IHC-P: 1:400 |

**Table S6. ssRNA probes used for RNA pull-down assay**

***COL3A1***

| Oligonucleotides name | Sequence (5’‒3’) |
| --- | --- |
| A probe | UGGUGUGGACGUUGGCCCUGUUUGCUUUUUAUAAACCAAACUCUAUCUGA‒Biotin |
| m^6^A probe 1 | UGGUGUGGACGUUGGCCCUGUUUGCUUUUUAUAAACCAAm^6^ACUCUAUCUGA‒Biotin |
| m^6^A probe 2 | UGGUGUGGACGUUGGCCCUGUUUGCUUUUUAUAAm^6^ACCAAACUCUAUCUGA‒Biotin |
| UTR NC probe | AAUUUAAAGAAAUAUUUUUAAAGCCACAAUUAUUUUAAUAUUGGAUAUCA‒Biotin |

***COL1A1***

| Oligonucleotides name | Sequence (5’‒3’) |
| --- | --- |
| A probe | AUGGAGGGAGUUUACAGGAAGCAGACAGGGCCAACGUCGAAGCCGAAUUC‒Biotin |
| m^6^A probe | AUGGAGGGAGUUUACAGGAAGCAGm^6^ACAGGGCCAACGUCGAAGCCGAAUUC‒Biotin |
| UTR NC probe | UACCCCCGCAUGGGUCUUCAAGCAAGUGGACCAAGCUUCCUUUUUUAAAA‒Biotin |

***ELN***

| Oligonucleotides name | Sequence (5’‒3’) |
| --- | --- |
| A probe | UCUGGGGCGCUUUUGGGUUGGAAAACCACCCCACACUGGGAAUAGCCACC‒Biotin |
| m^6^A probe | UCUGGGGCGCUUUUGGGUUGGAAAm^6^ACCACCCCACACUGGGAAUAGCCACC‒Biotin |
| UTR NC probe | AAUCACUCUAAUAUAACUCUGGAUGAAACACACCUUUUUUUUUAAUAAGA‒Biotin |

**Table S7. Primers used for ALKBH5 overexpression plasmid**

| Oligonucleotides name | Sequence (5’‒3’) |
| --- | --- |
| ALKBH5‒forward | AGAGCTAGCGAATTCATGGCGGCCGCCAGC |
| ALKBH5‒reverse | AAGATGCGGCGGCACGGATCCGACTACAAAGACC |
