# Supplementary material for: ALKBH5‐mediated m6A demethylation ameliorates extracellular matrix deposition in cutaneous pathological fibrosis
Source: Clin Transl Med. 2024 Sep 4;14(9):e70016. doi: 10.1002/ctm2.70016 (PMC11374695; doi:10.1002/ctm2.70016)
Supplement: Supplementary file 3 — Supporting Information [file CTM2-14-e70016-s002.docx]

**Supporting methods**

**Cell culture and treatment**

Tissue specimens obtained during surgery were dissected into 5 mm×5 mm sections and immersed in 0.3% dispase II (0.3 g/ml; Gibco, 17105041) at 4°C for 12 h. Then, the epidermal layer was removed, and the dermis was finely minced before incubation in collagenase NB4 (3 mg/ml; Nordmark, S1745401) at 37°C for 4 h to isolate dermal fibroblasts. Fibroblasts were isolated and cultured in Dulbecco’s Modified Eagle Medium (DMEM; Gibco, USA), supplemented with 10% fetal bovine serum (FBS; Gibco, USA) and 1% penicillin-streptomycin (Gibco, USA). Cultures were maintained at 37°C in a humidified atmosphere containing 5% CO2. Fibroblast adhesion was typically observed around 4–5 days post-culture initiation, with the cells reaching approximately 90% confluence after an additional 48 hours. Fibroblasts were passaged at a 1:3 ratio. Subsequent passages were conducted every 72 h for a maximum of six passages, adhering to standard protocols.

STM2457 was purchased from MedChemExpress (cat. HY-134836). Recombinant human TGF-β1 was obtained from MCE Med-ChemExpress (HY-P70543, Monmouth Junction, NJ, USA). And HDFs were treated with 10ng/mL recombinant human TGF-β1 for 24 hours.

**siRNA-mediated gene silencing**

Knockdown of *ALKBH5* and *YTHDF1* in HDFs was accomplished by transfection with siRNA sequences synthesized by Zorin Biotechnology Co., Ltd. (Shanghai, China). The specific siRNA sequences used are listed in Table S3. The transfection procedure was carried out using Lipofectamine 3000 transfection reagent (Thermo Fisher, USA) according to the manufacturer’s instructions. Briefly, a total of 200 μl of DMEM was mixed with 5 μl of Lipofectamine 3000 and 5 μl of 20 μM siRNAs or scrambled control RNA. After 20 min of incubation, the solution was added to 1.8 ml of DMEM and transferred to fibroblasts seeded in 6-well plates. RNA and protein samples were extracted 48-72 h after transfection for further experimentation.

**Overexpression plasmid construction, lentiviral packaging and transfection**

For the overexpression of ALKBH5 in HSFs, overexpression and negative control plasmids with marked GFP were synthesized by Zorin Biotechnology Co., Ltd. (Shanghai, China). The primers used for constructing the overexpression vectors are detailed in Table S7. Subsequently, a lentiviral packaging kit (Zorin, China) was used to generate lentiviruses in 293T cells according to the manufacturer’s instructions. The virus-containing supernatant was collected 48 and 72 h post-transfection and filtered through a 0.45-μm cellulose acetate filter. For transduction, HSFs, which had been seeded 24 hours prior, were exposed to medium containing 25 μl/ml of concentrated lentivirus and 5 ng/ml polybrene. The cells were maintained in this virus-containing medium for 48 hours. To select for stable ALKBH5 overexpression, HSFs were treated with 4 μg/ml puromycin for 2 weeks and subsequently maintained in medium containing 1 μg/ml puromycin.

**RNA extraction, reverse transcription and quantitative real-time PCR (qRT-PCR)**

Total RNA was extracted using TRIzol reagent (Solarbio, USA). Reverse transcription was performed using the PrimeScript RT Master Mix (Takara, RR036A). Quantitative real-time PCR (qRT-PCR) was conducted on an ABI QuantStudio 6 Flex system with SYBR Premix (Takara, RR066A), following the manufacturer’s instructions. The primer sequences utilized are listed in Table S4.

**WB and immunofluorescence analysis**

Tissues and cultured cells were lysed for 30 min with RIPA lysis buffer supplemented with protease inhibitor (Roche, Mannheim, Germany). For inducible protein expression analysis, 20 μg of protein was resolved by 10% sodium dodecyl sulphate-polyacrylamide gel electrophoresis (SDS-PAGE) and transferred onto polyvinylidene difluoride (PVDF) membranes (Merck-Millipore, Shanghai, China). Membranes were blocked with 5% non-fat milk for 1 hour at room temperature, followed by overnight incubation with primary antibodies at 4°C. Afterward, the membranes were incubated with peroxidase-conjugated secondary antibody for 1 h at room temperature and developed using enhanced chemiluminescence (ECL) western blotting detection reagent (Millipore, Billerica, MA). Immunoreactive bands were quantitatively analysed using Image J software.

For immunofluorescence staining, paraffin-embedded tissue sections were deparaffinized, rehydrated, fixed, and blocked with 5% normal goat serum. Cultured cells were directly fixed in 4% paraformaldehyde and subsequently blocked. Both tissue sections and cells were then incubated with primary antibodies overnight at 4°C. The following day, tissue slides were treated with secondary antibodies for 60 minutes, and nuclei were counterstained with 4',6-diamidino-2-phenylindole (DAPI, Sigma-Aldrich, St. Louis, MO, USA) for 5 minutes. Fluorescence analysis was conducted using a Zeiss 710 laser-scanning microscope (Zeiss, Oberkochen, Germany). Analysis of immunofluorescence colocalization and the signal intensity of individual channels was performed using Image J software.

The antibodies used for WB and immunofluorescence analyses are listed in Table S5.

**Cell proliferation assay**

Cell proliferation was evaluated using a CCK-8 kit (Dojindo, Tokyo, Japan) following the manufacturer’s protocols. In brief, cells were plated in triplicate in 96-well plates at a density of 2000 cells/100 μl. At the specified time points, CCK-8 solution was added to the wells, and the plates were incubated at 37 °C for 3-4 h before the absorbance was measured at 450 nm. Additionally, cell proliferation was assessed using a 5-ethynyl-2′-deoxyuridine (EdU) DNA Cell Proliferation Kit (Beyotime Biotechnology, China) following the manufacturer’s protocol. The proportion of cells incorporating EdU was analysed using a Zeiss 710 laser-scanning microscope (Zeiss, Oberkochen, Germany).

**Cell apoptosis and cell cycle assay**

Apoptosis was assessed using a FITC-Annexin V Apoptosis Detection Kit 1 (BD Biosciences, San Diego, CA) following the manufacturer’s instructions. Specifically, the cells were washed twice with cold PBS and then stained with FITC-Annexin V and PI on ice for 5 min. For cell cycle analysis, approximately 10^6^ cells were collected and fixed with 75% cold ethanol at 4°C for 2 h. Subsequently, the fixed cells were stained with RNase A and propidium iodide (Cell Cycle Assay Kit, Dojindo, Japan) as per the manufacturer’s instructions. Flow cytometry analysis was performed using a BD LSRFortessa analyser (BD Biosciences).

**Transwell assay**

A 24-well Transwell system with polycarbonate filters (8-μm pores, Corning, NY, USA) was used for this assay. In the upper compartment, 10,000 cells were suspended in medium supplemented with 2% FBS, while the lower chamber contained medium with 10% FBS. After a 1-day incubation at 37°C, the cells in the Transwell system were stained with 0.25% crystal violet. Cells remaining in the upper chamber were removed, and those that migrated to the lower chamber were photographed and quantified.

**Wound healing assay**

HDFs were seeded into 6-well plates and transfected with siRNA. Once the cells reached confluence, a sterile 1-ml pipette tip was used to create a scratch across the monolayer, and debris was removed by washing. The cells were then cultured at 37°C in a 5% CO_2_ atmosphere. Images were captured at 0, 24, 48 and 72 h using an inverted microscope (Nikon, Japan). Images of the wound area were captured at 0, 24, 48, and 72 hours using an inverted microscope (Nikon, Japan). Wound areas were quantified using ImageJ software and normalized to the initial wound area at 0 hours.

**RNA sequencing and data analysis**

Total RNA was extracted from cultured HDFs transfected with siRNA using TRIzol reagent (Solarbio, China). Eukaryotic mRNA was enriched using Poly-T oligo-attached magnetic beads. After fragmentation, the mRNA was converted into individual cDNA libraries. Following cluster generation, the libraries were sequenced on an Illumina NovaSeqTM 6000 platform. Gene expression levels were quantified as fragments per kilobase of exon model per million mapped reads (FPKM). Differentially expressed genes (DEGs) were identified using the DESeq2 algorithm, with thresholds set at a false discovery rate (FDR) < 0.05 and | log2(fold change) | ≥1. The RNA-seq data were deposited in the GEO database (GSE264515). Sequencing was conducted by Kangcheng Biotech, Inc. (Shanghai, China).

GO analysis of the designated genes was performed using DAVID (http://david.abcc. ncifcrf.gov/). Fisher’s exact test was employed to identify the significant GO categories, with FDR applied for P value correction. GO terms with P < 0.05 were considered significant. Enrichment maps were created using Cytoscape 3.7.0, and bubble plots were constructed using GraphPad Prism 9.0 (GraphPad Software, Inc.), where each node represents a GO pathway, and the node size is proportional to the total number of genes in each pathway.
